# Topics, Trends, and Sentiments of Tweets About the COVID-19 Pandemic: Temporal Infoveillance Study

**DOI:** 10.2196/22624

**Published:** 2020-10-23

**Authors:** Ranganathan Chandrasekaran, Vikalp Mehta, Tejali Valkunde, Evangelos Moustakas

**Affiliations:** 1 Department of Information and Decision Sciences University of Illinois at Chicago Chicago, IL United States; 2 Middlesex University Dubai Dubai United Arab Emirates

**Keywords:** coronavirus, infodemiology, infoveillance, infodemic, twitter, COVID-19, social media, sentiment analysis, trends, topic modeling, disease surveillance

## Abstract

**Background:**

With restrictions on movement and stay-at-home orders in place due to the COVID-19 pandemic, social media platforms such as Twitter have become an outlet for users to express their concerns, opinions, and feelings about the pandemic. Individuals, health agencies, and governments are using Twitter to communicate about COVID-19.

**Objective:**

The aims of this study were to examine key themes and topics of English-language COVID-19–related tweets posted by individuals and to explore the trends and variations in how the COVID-19–related tweets, key topics, and associated sentiments changed over a period of time from before to after the disease was declared a pandemic.

**Methods:**

Building on the emergent stream of studies examining COVID-19–related tweets in English, we performed a temporal assessment covering the time period from January 1 to May 9, 2020, and examined variations in tweet topics and sentiment scores to uncover key trends. Combining data from two publicly available COVID-19 tweet data sets with those obtained in our own search, we compiled a data set of 13.9 million English-language COVID-19–related tweets posted by individuals. We use guided latent Dirichlet allocation (LDA) to infer themes and topics underlying the tweets, and we used VADER (Valence Aware Dictionary and sEntiment Reasoner) sentiment analysis to compute sentiment scores and examine weekly trends for 17 weeks.

**Results:**

Topic modeling yielded 26 topics, which were grouped into 10 broader themes underlying the COVID-19–related tweets. Of the 13,937,906 examined tweets, 2,858,316 (20.51%) were about the impact of COVID-19 on the economy and markets, followed by spread and growth in cases (2,154,065, 15.45%), treatment and recovery (1,831,339, 13.14%), impact on the health care sector (1,588,499, 11.40%), and governments response (1,559,591, 11.19%). Average compound sentiment scores were found to be negative throughout the examined time period for the topics of spread and growth of cases, symptoms, racism, source of the outbreak, and political impact of COVID-19. In contrast, we saw a reversal of sentiments from negative to positive for prevention, impact on the economy and markets, government response, impact on the health care industry, and treatment and recovery.

**Conclusions:**

Identification of dominant themes, topics, sentiments, and changing trends in tweets about the COVID-19 pandemic can help governments, health care agencies, and policy makers frame appropriate responses to prevent and control the spread of the pandemic.

## Introduction

As the effects of the COVID-19 pandemic are felt worldwide, social media platforms are becoming inundated with content associated with the disease. Since its initial identification and reporting in Wuhan, China, the novel disease COVID-19 has spread to multiple countries across all continents and has become a global pandemic. The World Health Organization (WHO) declared the outbreak to be a pandemic on March 11, 2020, and the US government declared it to be a national emergency on March 13, 2020. As of June 30, 2020, the virus has infected over 10 million individuals and has caused approximately 503,000 deaths worldwide [1]. To contain the spread of the virus, several countries have implemented lockdown and quarantine measures and imposed travel bans, restricting people’s movement. Schools have been closed, many workers have become unemployed, and numerous individuals are locked down in their homes. With millions of lives affected by the COVID-19 pandemic, social media platforms such as Twitter have become an outlet for users to express their concerns, opinions, and feelings about the pandemic.

Social media has emerged as a significant conduit for health-related information; the majority of people across multiple countries use some form of social media [1,2]. Pew Research surveys examining multiple countries have identified social media as an important source of health information [3]. In recent years, sharing and consuming health information via social media has become prevalent. It is unsurprising that social media has become a prominent platform for people to share information and feelings about COVID-19.

The science of understanding health-related information that is distributed via a digital medium such as the internet or social media with the aim to inform public health and public policy is known as infodemiology. A related term, infoveillance, refers to syndromic surveillance of public health-related concerns that is expressed and diffused on the internet through digital channels. Infoveillance has been particularly useful to identify outbreak patterns and to study public perceptions of several diseases, including H1N1 influenza (“swine flu”) [4], Ebola virus [5,6], and Zika virus [7-9]. Analysis of health event data posted on social media platforms not only provides firsthand evidence of health event occurrences but also enables faster access to real-time information that can help health professionals and policy makers frame appropriate responses to health-related events.

The COVID-19 outbreak has propelled an emergent set of studies that have examined public perceptions, thoughts, and concerns about this pandemic using social media data (Table 1). Most of these studies relied on data from the Twitter or Weibo platforms and analyzed data from early periods of the pandemic. The amount of data used in these studies varies from a few hundred tweets to a few million. These studies have collectively provided a rich body of knowledge on how Twitter users have reacted to the pandemic and their concerns in the early stages of the outbreak. Many of these studies did not differentiate between sources of tweets, such as whether the tweet originated from an individual or an organization such as a news channel or health agency. From an infoveillance perspective, it is important to understand the social media discourses pertaining to COVID-19 among the common public rather than by news agencies or other organizations. Further, there is limited understanding of the changes in public sentiments and discourse about COVID-19 over time. To address these gaps, we examined COVID-19–related tweets using a much larger data set covering a time period from January 1 to May 9, 2020. We performed a temporal assessment and examined variations in the topics and sentiment scores over a period of time from before to after the disease was declared a pandemic to uncover key trends.

Our research goals were to examine key themes and topics in COVID-19–related English-language tweets posted by individuals and to explore the trends and variations in how COVID-19–related tweets, key topics, and associated sentiments changed over a period of time from before to after the disease was declared a pandemic.

**Table 1:** Summary of key studies on the COVID-19 pandemic using social media data.

Source	Social media platform	Data set	Time period	Key findings
Abd-Alrazaq et al, 2020 [10]	Twitter	167,073 tweets	Tweets from February 2 to March 15, 2020	Identified 12 topics that were grouped into four themes, viz the origin of the virus; its sources; its impact on people, countries, and the economy; and ways of mitigating infection.
Li et al, 2020 [11]	Weibo	115,299 posts	Posts from December 23, 2019, to January 30, 2020	Positive correlation between the number of Weibo posts and number of reported cases in Wuhan. Qualitative analysis of 11,893 posts revealed main themes of disease causes, changing epidemiological characteristics, and public reaction to outbreak control and response measures.
Shen et al, 2020 [12]	Weibo	15 million posts	Posts from November 1, 2019, to March 31, 2020	Developed a classifier to identify “sick posts” pertaining to COVID-19. The number of sick posts positively predicted the officially reported COVID-19 cases up to 14 days ahead of official statistics.
Sarker et al, 2020 [13]	Twitter	499,601 tweets from 305 users who self-disclosed their COVID-19 test results	N/A^a^	203 users who tested positive for COVID-19 reported their symptoms: fever/pyrexia, cough, body ache/pain, fatigue, headache, dyspnea, anosmia and ageusia.
Tao et al, 2020 [14]	Weibo	15,900 posts	December 31, 2019, to March 16, 2020	Analysis of oral health–related information posted on Weibo revealed home oral care and dental services to be the most common tweet topics.
Wahbeh et al, 2020 [15]	Twitter	10,096 tweets from 119 medical professionals	December 1, 2019, to April 1, 2020	Identified eight themes: actions and recommendations, fighting misinformation, information and knowledge, the health care system, symptoms and illness, immunity, testing, and infection and transmission.
Budhwani et al, 2020 [16]	Twitter	193,862 tweets by US-based users	March 9 to March 25, 2020	Identified a large increase in the number of tweets referencing “Chinese virus” or “China virus.”
Rufai and Bunce, 2020 [17]	Twitter	203 viral tweets by 8 G7^b^ world leaders	November 17, 2019, to March 17, 2020	Identified three categories of themes: informative, morale-boosting, and political.
Park et al, 2020 [18]	Twitter	43,832 users and 78,233 relationships	Few weeks before February 29, 2020	Assessed speed of information transmission in networks and found that news containing the word “coronavirus” spread faster.
Lwin et al, 2020 [19]	Twitter	20,325,929 tweets from 7,033,158 users	January 28 to April 9, 2020	An examination of four emotions (fear, anger, sadness, and joy) revealed that emotions shifted from fear to anger, while sadness and joy also surfaced.
Pobiruchin et al, 2020 [20]	Twitter	21,755,802 tweets from 4,809,842 users	February 9 to April 11, 2020	Examined temporal and geographical variations of COVID-19–related tweets, focusing on Europe, and the categories and origins of shared external resources.

^a^N/A: not applicable.

^b^G7: Group of Seven.

## Methods

### Data Collection

We collected all COVID-19–related tweets from January 1 to May 9, 2020. The Python programming language was used for our data collection and analyses, and Tableau was used as a supplementary tool for visualization purposes. We used three sources to assemble the tweets required for our analysis. First, we relied on the COVID-19 Twitter data set at IEEE Dataport [21], which contained COVID-19–related tweets from March 20, 2020. Second, we used a Twitter data set posted in GitHub [22] that contained COVID-19–related tweets posted since January 21, 2020. Both these data sets are publicly available and provide a list of tweet IDs for all tweets related to COVID-19. Third, we collected COVID-19–related tweets for the remaining period, including texts and metadata, from Twitter using GetOldTweets3, a Python 3 library that enables scraping of historical Twitter data [23]. Because we were combining tweets from multiple sources, we used a common set of keywords and phrases that other sources had used: *corona*, *coronavirus*, *covid-19*, *covid19*, and their variants, including their hashtag equivalents. The language-tag setting “EN” and the retweet tag “RT” were used to filter English-language tweets and retweets. We also used the retweets feature in GetOldTweets3 to filter out retweets. Due to restrictions of the Twitter platform, the public data sets contained only the tweet IDs. The process of extracting complete details of a tweet, including metadata, from Twitter using the tweet ID is referred to as hydration, and a number of tools have been developed for this purpose [11]. We used the Hydrator software [24] listed in IEEE Dataport to gather the complete text and metadata of the tweets.

### Data Preprocessing

Our next step was to classify all the tweets posted by individuals versus those that originated from organizations. We first gathered the unique Twitter user IDs of all the Twitter users in our data set. Following the approach outlined in [25], we used a naïve Bayes machine learning model to classify the tweeters into individuals versus organizations. We used a published data set that contained 8945 Twitter users and their profile descriptions, which human coders used to annotate users as individual or institutional [26]. To these data, we added 2000 Twitter user IDs pulled from our data set along with their associated profiles, and we manually annotated them (interrater reliability κ=0.84). Using the combined data set of 10,945 users, we divided our data set into training versus validation sets using an 80:20 split, and we used these sets to train and test our classifier model, respectively. The naïve Bayes classifier yielded an accuracy of 83.2% with a precision of 0.82, a recall of 0.83, and an F1 score of 0.81; these values were considered satisfactory and are comparable to those in other studies [27-29]. Multimedia Appendix 1 presents the confusion matrix. Our classifier performance was also robust across multiple split strategies for dividing the data set for training and validation. This classifier was then used to identify all the individual users in our full data set, and only tweets posted by individuals were retained for further assessment. We also eliminated duplicate tweets and retweets (filtered using the “RT” tag), resulting in a data set that contained only original tweets posted by individual users. We preprocessed and cleaned the tweets using the Natural Language Toolkit (NLTK), regular expression (RegEx), and the gensim Python library [30]. We removed stop words, user mentions, and links, and we also lemmatized the text of the tweets.

### Topic Modeling and Sentiment Analysis

Topic modeling is an unsupervised machine learning approach that is useful for discovering abstract topics that occur in a collection of textual documents. It helps uncover hidden semantic structures in a body of documents. Most topic modeling algorithms are based on probabilistic generative models that specify mechanisms for how documents are written in order to infer abstract topics. One popular topic modeling algorithm, latent Dirichlet allocation (LDA), is an unsupervised generative probabilistic method for modeling a corpus of words [31]. A key advantage of LDA is that no prior knowledge of topics is needed. By tuning the LDA parameters, one can explore the formation of different topics and the resultant document clusters. Despite the usefulness of LDA, its outcomes can be difficult to interpret and can drastically vary based on the choice of parameters. With a large corpus of texts, the unsupervised nature of LDA can result in the generation of topics that are neither meaningful nor effective, requiring human intervention and multiple iterations [10]. An improvised variant to traditional LDA, the guided LDA algorithm [32], enables the provision of a set of seed words that are representative of the underlying topics so that the topic models are guided to learn topics that are of specific interest.

We used two broad approaches to prepare the initial set of topics and the seed words for guided LDA. First, we used the extant literature on COVID-19 infoveillance using Twitter to identify a broad set of topics and potential keywords. Second, we performed traditional LDA with multiple numbers of topics as inputs (n=10, 20, 30, and 40) iteratively and examined the word lists that were generated. We used both steps to generate a list of topics and anchor words for the guided LDA (see Multimedia Appendix 2). The GuidedLDA package in Python was used for the topic modeling. Through discussions, the authors then grouped the topics and identified dominant themes. Further, we computed a sentiment score for each tweet using the VADER (Valence Aware Dictionary and sEntiment Reasoner) tool in Python. VADER is a lexicon and rule-based sentiment analysis tool that is specifically attuned to sentiments in social media texts such as tweets [33].

To assess the sentiments of tweets, VADER provides a compound score metric that calculates the sum of all Lexicon ratings that have been normalized between –1 (most extreme negative) and +1 (most extreme positive); this method takes into account both the polarity (positive/negative) and the intensity of the emotion expressed. For each tweet, we classified the sentiment as positive, negative, or neutral based on the compound score. A tweet with a compound score greater than 0.05 was classified as positive, a tweet with a score between –0.05 and 0.05 was classified as neutral, and a tweet with a score less than –0.05 was classified as negative. To further understand the changes in the sentiment scores over time, we qualitatively analyzed the content of tweets to explore the rationale behind the changes in the compound sentiment scores. The authors manually examined the tweets pertaining to specific topics in weeks in which variations were observed to infer possible reasons for the variations in sentiment.

## Results

We obtained a total of 13,937,906 tweets from 10,868,921 unique users after eliminating 4,085,264 tweets posted by organizations and institutions. Our primary goal was to understand public perceptions and sentiments pertaining to COVID-19; hence, only tweets posted by individuals were retained for analysis.

### Themes and Topics From Text Mining

Our analysis of tweets yielded 26 subtopics, which we framed into 10 broad themes (Table 2). Of the 13,937,906 tweets we examined, 2,858,316 (20.51%) pertained to the theme of the impact of COVID-19 on the economy and markets, followed by spread and growth in cases (2,154,065, 15.45%), treatment and recovery (1,831,339, 13.14%), impact on the health care sector (1,588,499, 11.40%), and government response to the pandemic (1,559,591, 11.19%). Although tweets related to the theme of racism formed only 4.14% (577,066/13,937,906) of the data, over 500,000 tweets were found to contain racist content. It should be noted that all the tweets we assessed were public discourses pertaining to broader themes, as our data set consisted of tweets posted by individuals about various issues pertaining to the COVID-19 pandemic.

**Table 2:** Themes and topics of COVID-19–related tweets (N=13,937,906), n (%).

Theme and topics		Value
**1. Source (origin)**		**966,372 (6.93)**
	1.1 Outbreak	489,768 (3.51)
	1.2 Alternative causes	476,606 (3.42)
**2. Prevention**		**1,076,840 (7.73)**
	2.1 Social distancing	575,786 (4.13)
	2.2 Disinfecting and cleanliness	501,054 (3.59)
	3. Symptoms	558,332 (4.01)
**4. Spread and growth**		**2,154,065 (15.45)**
	4.1 Modes of transmission	472,749 (3.39)
	4.2 Spread of cases	617,946 (4.43)
	4.3 Hotspots and locations	459,039 (3.29)
	4.4 Death reports	604,331 (4.34)
**5. Treatment and recovery**		**1,831,339 (13.14)**
	5.1 Drugs and vaccines	442,413 (3.17)
	5.2 Therapies	483,109 (3.47)
	5.3 Alternative methods	416,530 (2.99)
	5.4 Testing	489,287 (3.51)
**6. Impact on the economy and markets**		**2,858,316 (20.51)**
	6.1 Shortage of products	513,703 (3.69)
	6.2 Panic buying	667,320 (4.79)
	6.3 Stock markets	535,262 (3.84)
	6.4 Employment	505,510 (3.63)
	6.5 Impact on business	636,521 (4.57)
**7. Impact on health care sector**		**1,588,499 (11.4)**
	7.1 Impact on hospitals and clinics	441,895 (3.17)
	7.2 Policy changes	615,027 (4.41)
	7.3 Frontline workers	531,577 (3.81)
**8. Government response**		**1,559,591 (11.19)**
	8.1 Travel restrictions	519,406 (3.73)
	8.2 Financial measures	485,277 (3.48)
	8.3 Lockdown regulations	554,908 (3.98)
9. Political impact		767,486 (5.51)
10. Racism		577,066 (4.14)

### Trends in the Proportions of Positive, Negative, and Neutral COVID-19 Tweets

For each theme pertaining to COVID-19, we examined the trends in the proportions of positive, negative, and neutral tweets over time (Figure S1 in Multimedia Appendix 3). Of the total tweets concerning the source of the COVID-19 outbreak, the proportions of neutral and negative tweets remained fairly high (approximately 35% to 45%) in the weeks before the WHO announced that COVID-19 was a pandemic. The proportion of positive tweets exceeded those of negative and neutral tweets in the week of the WHO declaration. In the subsequent weeks, the proportion of positive tweets dropped to approximately 25%, whereas the proportions of neutral and negative tweets were approximately 30% to 45%. When we examined tweets pertaining to the prevention of COVID-19, the proportion of positive tweets exceeded those of neutral and negative tweets in almost all the weeks from February 2020, reaching approximately 40% in the beginning of May 2020.

The proportion of negative tweets was considerably higher than those of the positive and neutral tweets for the themes of symptoms (approximately 60%) and of spread and growth in cases (approximately 45%). This pattern was observed for almost all the weeks we examined. In February 2020, over 90% of tweets on the theme of symptoms were negative. Although this trend gradually declined over the next few weeks, it still formed over 50% in the last week of our examination. Similarly, negative tweets about the spread and increase in COVID-19 cases constituted between 40% and 50% from February 2020 until the beginning of May 2020. For the theme of treatment and recovery, the proportion of positive tweets (20%) gradually increased to over 40% over the 17-week period. The negative tweets in the initial weeks (30% to 35%) declined to 25% in April and early May 2020.

We noted a gradual increase in the proportion of positive tweets pertaining to the impact of COVID-19 on the economy and markets over time. Proportions of negative tweets were higher in the months of February and March 2020 but gradually declined to approximately 30% toward the beginning of May 2020.

An increase in the proportion of positive tweets over time was seen for the themes of government response and impact on the health care industry. The theme pertaining to government response captured the Twitter discourse by users concerning various measures taken by different governments to address COVID-19. The proportion of negative tweets about government response was approximately 45% up to mid-March 2020 and then declined to approximately 30% by the first week of May. The proportion of negative tweets on the theme of the political impacts of COVID-19 was considerably higher (>50%) from March 2020. We also noted a substantial proportion of negative tweets on the theme of racism*.*

### Trends in Sentiments of Themes of COVID-19 Tweets

We examined the trends pertaining to the changes in the sentiment scores of each of our themes and topics over the time period of examination. To plot the trends, we used the average compound scores by topic and week. Our results are presented in Figure S2 (Multimedia Appendix 3).

Average compound sentiment scores were found to be negative throughout the time period of our examination for the themes of spread and growth of cases, symptoms, racism, source of the outbreak, and political impacts of COVID-19. In contrast, we saw a reversal of sentiments from negative to positive for the themes of prevention, impact on the economy and markets, government response, impact on the health care industry, and treatment and recovery; the negative sentiment scores in the initial weeks of the COVID-19 outbreak for the aforementioned themes changed to positive scores in the final few weeks of our examination. This reversal of sentiments is noteworthy, as it reflects a collective opinion of a fairly larger set of Twitter users on how the pandemic is being managed by key stakeholders.

### Trends in Sentiments of Topics of COVID-19 Tweets

We further examined the trends in the sentiment scores for topics underlying the broader themes. Compound scores from VADER were averaged over each topic for every week (Figure S3, Multimedia Appendix 3). This assessment helped us to understand the progression of sentiments for specific topics over the period we examined. To understand the variations in the sentiments, we also qualitatively examined the tweets for weeks in which changes were observed. Sample tweets for each of the themes and topics are shown in Multimedia Appendix 4.

Our analysis revealed a consistently negative average compound score for the topic of the outbreak in Wuhan, China, for all the weeks that we examined. We found that Twitter users frequently referred to the geographical origin of the disease even in the later weeks of our examination. When we examined the topic of alternative causes of the outbreak, we found several tweets about hypothetical causes and conspiracy theories pertaining to COVID-19 (eg, use of SARS-CoV-2 as a bioweapon and origin of the virus in a lab in Wuhan). The average sentiment scores remained negative for weeks until the week of March 22 to 28, then showed a spike to positive values and continued to remain positive until the beginning of May 2020. This positive trend in later weeks is due to tweets dismissing the conspiracy theories that were circulated during the early weeks. Further, the spike in the positive score in the week of March 22 to 28 is partly due to a large number of tweets that contained references to “coronavirus as an act of God” and prayers to end the pandemic, as well as tweets that viewed COVID-19 as “nature’s way to heal the planet.” These types of tweets provide qualitative evidence for the positive sentiment scores we observed in the analysis, such as:

This virus is certainly God’s call to humanity to wake up and recognise him before it is too late.

Wow. Earth is recovering, Air pollution is slowing down, Water pollution is clearing up, Natural wildlife is returning home, Coronavirus is earth’s vaccine. We’re the virus.

This planet will surely heal, in the most magical ways. I can feel the vibrations coming on.

The average compound score for social distancing remained negative until the first week of March. During this time period, COVID-19 had not yet spread worldwide. However, from the second week of March 2020, the average sentiment score was positive for all the weeks we examined, reflecting that the general public supported and had a favorable disposition toward social distancing as a mechanism to combat the spread of the virus. We observed that several Twitter appealed to others and advocated social distancing measures, such as:

Kindly stay at home. Wash your hands. Practice social distancing.

Ran two miles even when I didn’t want to! Made excuses all day! Get out there and do it! But practice social distancing, let’s flatten this curve!

The topic of disinfecting and cleanliness showed average positive sentiment scores for all weeks from the third week of January 2020. We found that Twitter users used gaming strategies such as challenges (eg, #SafeHandsChallenge) involving a chain of users to advocate cleanliness and create broader awareness about the importance of disinfecting and cleanliness. We found that many Twitter users shared tips about disinfecting groceries and products after shopping. We also found that some Twitter users condemned people who did not wear face masks or follow recommended safety protocols in public.

Three of the four topics under the theme of spread and growth exhibited negative average compound sentiment scores. The average compound scores for the topic of death reports were negative for all the weeks, with values ranging between –0.2 and –0.5. The topic pertaining to spread of cases exhibited negative trends throughout, with average compound scores ranging between –0.1 and –0.3. The topic of modes of transmission of COVID-19 also showed negative scores across all the weeks, with values between 0 and –0.2. The topic of hotspots and locations for COVID-19 transmission exhibited negative scores until February 2020 but showed positive scores thereafter. Tweets mentioning hotspots of COVID-19 transmission often included mentions of places such as churches, places of religious worship, beaches, events, and festive occasions with mass gatherings; these mentions primarily contributed to the positive values.

All four topics under the theme of treatment and recovery showed negative scores in the initial weeks, which changed to positive average compound scores from April 2020. For the topic of testing, Twitter users reacted negatively to the lack of availability of test kits and testing methods in the initial weeks of the pandemic (eg, tweets containing phrases such as “not all in the hospitals can be tested as they are often short with test kits” or “Many places are not testing people for coronavirus due to test shortage. Its annoying”); however, with the improvement in availability of COVID-19 test kits and test centers worldwide, the sentiment became positive:

We got tested today. Easy as could be, no waiting, felt really safe, cheek swabs.

In lots of states, it’s very easy to get tested now, even if you’re asymptomatic.

As more information on the efficacies of drugs such as remdesivir became available, Twitter users’ sentiments regarding drugs and medicines for COVID-19 became positive by the end of March 2020. Twitter users reacted to news about drugs, as can been seen in these example tweets:

HCQ and Remdesivir, are effective at limiting duration of illness, hospitalization and viral spread if given early.

Remdesivir is effective in mitigating COVID-19 symptoms if taken early, ideally pre-hospitalization.

We also noted a small increase in the average positive compound score for the topic of drugs and medicines in the last week of April; this can be attributed to the US Food and Drug Administration’s authorization of emergency use of the antimalarial drug chloroquine to treat COVID-19 on April 27, 2020. Tweets such as these contributed to this positive compound score:

Hydroxychloroquine protocol: effective, cheap and can be produced in many laboratories. HCQ functions as both a cure and a vaccine.

However, it should be noted that this authorization was later revoked on June 15, 2020, a date outside the time period covered by our study. The negative sentiments about various therapies in the initial weeks of the pandemic also started to become positive in the third week of March. Twitter users positively reacted and shared information on plasma therapy and associated trials that were being conducted on patients with COVID-19. For instance, some tweets provided examples of positive sentiments from Twitter users:

We desperately need a treatment for those severely suffering with Coronavirus. Blood plasma could be the answer.

The effects of coronavirus are scary for many families, but this treatment of using antibodies from recovered patients could save lives.

The topic of alternative methods of treatment for COVID-19 (traditional Chinese medicine, Indian Ayurveda, etc.) had mildly positive sentiment scores for most of the time period in our examination.

Among the topics that comprise the theme of impact on the economy and markets, Twitter users’ average compound scores for sentiments about the topic of employment remained negative for all the weeks we examined. Many Twitter users posted information about their job loss and unemployment:

Lost my job a couple weeks ago due to Coronavirus and now it’s impossible to find a new job.

Well....I just got the call. Lost my job due to Covid.

Moreover, other users organized crowdfunding campaigns to help people who lost their jobs. Similar negative scores were seen across all the weeks for the topic pertaining to stock markets. Tweets pertaining to the topic of panic buying by consumers showed negative sentiment scores for all the weeks until mid-April, after which the scores began to be positive. Many users shared tweets about long queues and panic buying, as can be seen in these tweets:

2020 and panic buying has reduced us to this. Waiting in line for at least 2 hours to get pull ups and baby wipes because no one else has them.

No panic buying y’all hear that? So leave some damn bread and milk for me please.

Tweets about the topic of shortages (of food and essential items) swung between positive and negative scores in the first few weeks of the pandemic but became positive from mid-March until early May. Twitter users reacted positively to measures adopted by supermarkets and grocery stores to practice safety measures, as can be seen in this illustrative tweet:

Yes! Longos Markets requires all customers to wear masks. Went there today, it was a good, safe shopping experience, better than any other store. Will definitely be shopping there again.

Twitter users’ sentiments about the topic of businesses exhibited negative scores in the initial weeks of the pandemic, primarily fueled by news about closures and losses. However, this sentiment score changed to positive in the first week of March 2020. The positive score seen here reflects the adaptation of businesses to the new pandemic and their reopening and revival across different countries. Tweets such as the following provide indicators of positive sentiments of users about reopening of businesses:

Most of HK open for business now. Emphasis on testing and tracing.

Open for business. Trusting the people to take care of themselves. Freedom smells sweet.

Sentiment scores pertaining to the topic of hospitals and clinics largely remained negative throughout the time period of our examination. Tweets about lack of beds, facilities, NS ventilators, overcrowding of patients, and the struggles of health care institutions to cater to the influx of COVID-19 patients contributed to the negative sentiment scores. Illustrative tweets about these negative sentiments include:

Madrid hospitals now have double the number of intensive care patients than beds. Means you can no longer get intensive care in a Madrid hospital.

Lack of safety gear for healthcare workers, shortage of beds and doctors, inadequate labs to conduct tests - our healthcare system is very fragile!!

However, we noted a reversal in the trends of sentiment scores for the topic of frontline health care workers. Twitter users’ negative sentiments until the end of March 2020, reflecting the lack of personal protective equipment and gear for health care professionals, health worker burnout, and increased workload for health care workers, became positive in April and early May 2020 as the situation improved. We saw tweets in the initial period of the pandemic with negative sentiments, such as “the knighted geniuses at the top of the NHS can’t even organise protective equipment for our doctors and nurses”. In the later weeks of our examination, hashtags such as #coronawarriors and tweets hailing the services of frontline workers (eg, “Deepest gratitude to the #CoronaWarriors who are working tirelessly in these difficult times*”*) contributed to positive sentiment scores. The topic of health policy, which reflects the newer safety guidelines, protocols, and policies pertaining to patients with COVID-19 implemented by health care organizations, exhibited a negative trend in the early period, with tweets such as:

The ventilator situation is even more dire than we know. Not every hospital had an allocation policy in place.

Spain has begun a no ventilator policy for anyone over 65.

However, this topic showed a positive trend after the end of March 2020 as many agencies, governments, and health care institutions began to establish clear policies for treating patients with COVID-19. In the later weeks of our examination, many hospitals had framed clearer guidelines for use of masks, visitations, and restrictions pertaining to COVID-19. These illustrative tweets point to a possible rationale behind the positive sentiments pertaining to the topic of health policy in the later weeks of our examination:

The hospital has an understandable policy during this crisis of limiting visitation for the safety of all & to reduce use of critical PPEs.

Spouse can’t visit under hospital’s no visitation policy. Psychologically excruciating but family all recognize it’s the right thing, and hard to limit exceptions once you start”

Twitter users’ sentiments about the topic of travel restrictions imposed by governments worldwide were largely negative for most of the weeks we examined, except for the week of March 22 to 28, 2020. In this week, governments in populous countries such as India and Canada announced travel restrictions such as flight suspensions and isolation and quarantine measures for individuals entering these countries. Tweets indicated positive sentiments about travel restrictions:

I believe it was a good move from India to have a complete travel restriction to all countries. When we don't have the health systems to treat huge populations, the best thing to do is to shut doors.

The fine for breaking self-quarantine / self-isolation in BC, Canada is $25,000 AND jail time. Canada is taking travel and quarantine very seriously. Great job.

Many Twitter users welcomed travel bans and restrictions and expressed positive sentiments about them.

Except for the first two weeks of April, the average compound sentiment scores about the topic of lockdown regulations remained negative in most of the weeks we examined. Twitter users’ sentiments about stay-at-home orders, shutdowns, and lockdowns of complete cities were negative. This can be seen from these sample tweets:

This lockdown really does do bad things to good peoples mental health, trying to stay positive is a task in itself when this shite feels never ending.

Lockdown extended for another 3 weeks I hate it here.

However, the sentiment scores were positive in the weeks in which different governments announced financial measures such as stimulus payments to people affected by closures and lockdowns. In some tweets, users expressed positive sentiments about the financial relief measures to help individuals suffering due to the impact of COVID-19:

I got my stimulus check today! Woohoo!

Zoe and I finally received our stimulus/relief check from the federal government.

India is preparing a stimulus package that would put money directly into the accounts of more than 100 million poor people and support businesses hit the hardest by the 21-day lockdown.

## Discussion

### Principal Findings

This study joins the growing body of infoveillance studies on COVID-19 that examine social media data to uncover public opinions about the pandemic. We used a corpus of over 13 million tweets from January until the first week of May 2020 to uncover the trends in sentiments regarding various themes and topics. Our study is comprehensive, covering 26 different topics underlying COVID-19–related tweets under 10 broader themes. In response to a call made by Liu et al [34], we combined the topic modeling approach with sentiment analysis to observe the trends in sentiments for various themes and topics over time. By assimilating the collective opinions of several million users, we found interesting patterns in the trends pertaining to sentiments of themes and topics of COVID-19–related tweets.

We combined two publicly available sources with our own search to assemble a unique data set that contained English-language tweets about various topics associated with COVID-19. We further used a naïve Bayes classifier to segregate tweets made by individuals. We employed guided LDA to identify the underlying topics and associated themes, and we also examined the sentiments associated with the tweets and their changes over time.

Our key finding is that the impact of COVID-19 on the economy and markets was the most discussed issue by Twitter users. The number and proportions of tweets on this theme were remarkably higher than those of tweets on the other themes we uncovered. Further, users’ sentiment was negative until the third week of March but gradually became positive in the final weeks we studied. Users’ initial negative sentiments about shortages, panic buying, and businesses gradually turned positive from April 2020. Users started feeling positive about government responses to contain the pandemic, including financial measures to support and assist them in dealing with the disease outbreak.

Twitter users felt negative about continued spread and growth of the number of cases and the symptoms of COVID-19. However, we also found that Twitter users were more positive about treatment of and recovery from COVID-19 in later weeks than they were during the earlier stages of the pandemic. They expressed positive feelings by sharing information on testing, drugs, and newer therapies that show promise to contain the outbreak. Another notable finding is the Twitter users’ gradual change in sentiment from negative to positive regarding COVID-19 prevention measures such as social distancing and cleanliness. Twitter users who initially expressed negative sentiments regarding COVID-19’s impacts on the health care sector, comprising hospitals, clinics, and frontline workers, gradually became positive in the later weeks.

Another key finding of our study is the continued negative sentiments about political fallouts due to COVID-19. Although leaders worldwide are struggling to contain the pandemic, we noted that Twitter users felt negative about how COVID-19 was used for political purposes. Similarly, we noted strong negative sentiments about racist content in users’ tweets.

Our study offers several insights for health policy makers, administrators, and officials who are managing the impact of the COVID-19 pandemic. Identification of topics and associated sentiment changes provide pointers to how the general public are reacting to specific measures taken to tackle the pandemic. Variations in sentiment scores serve as a feedback mechanism for assessing public perceptions of various measures taken with respect to social distancing, cleanliness and disinfecting, lockdowns, travel restrictions, and efforts to revive the economy. Public sentiments have also started to become positive about COVID-19 testing, treatment, and vaccines as well as health policies. Our study shows that observing aggregate sentiments and changes in trends via social media posts can offer a cost-effective, timely, and valuable mechanism to gauge public perceptions regarding policy decisions being made to address the pandemic.

### Limitations

This study has a few limitations that should be kept in mind while interpreting the results. We relied on a large data set that was partly compiled by us and included two other publicly available data sets. These data sources contained tweets from varying dates and used different search terms and search strategies to gather the tweets. Our analysis may have inadvertently omitted certain COVID-19–related tweets that were not captured by the data sources. In addition, COVID-19–related tweets from users who chose to make their accounts private were not included in our study. Further, we did not consider any geographical boundaries when examining the tweets. Studies focusing on tweets from specific countries can find different topics and sentiments that reflect country-specific opinions and concerns. We also restricted our study to tweets in the English language and to those posted by individuals. It should be noted that our naïve Bayes classifier with over 80% accuracy helped us identify tweets posted by individuals. It is possible that some individual users posted tweets on behalf of organizations, and these tweets may have been included in our data set. A more refined approach with deep learning techniques to identify individual tweets can aid in classifying and assembling a tweet data set with increased accuracy. As a future research extension, tweets posted by organizations could be another frame of reference to understand their concerns and sentiments. Another important limitation is that our findings are reflective of Twitter users, who are fairly familiar with social media and use of technology. The results may not generalize to the larger worldwide population of people who do not use Twitter.

### Conclusion

As COVID-19 continues to affect millions of people worldwide, our study throws light on dominant themes, topics, sentiments and changing trends regarding this pandemic among Twitter users. By examining the changing sentiments and trends surrounding various themes and topics, government agencies, health care organizations, businesses, and leaders who are working to address the COVID-19 pandemic can be informed about the larger public opinion regarding the disease and the measures they have taken so that adaptations and corrective courses of action can be applied to prevent and control the spread of COVID-19.

## Appendix

Multimedia Appendix 1 The confusion matrix.

Multimedia Appendix 2 Themes, topics, and associated keywords.

Multimedia Appendix 3 Supplementary figures showing the trends in the proportions of positive, neutral, and negative tweets, sentiment score trends by theme, and trends in sentiment scores by topic.

Multimedia Appendix 4 Illustrative tweets for the topics and themes.

## Abbreviations

LDA: latent Dirichlet allocation
NLTK: Natural Language Toolkit
RegEx: regular expression
VADER: Valence Aware Dictionary and sEntiment Reasoner
WHO: World Health Organization
