# Supplementary material for: Topics, Trends, and Sentiments of Tweets About the COVID-19 Pandemic: Temporal Infoveillance Study
Source: J Med Internet Res. 2020 Oct 23;22(10):e22624. doi: 10.2196/22624 (PMC7588259; doi:10.2196/22624)
Supplement: Multimedia Appendix 1 [file jmir_v22i10e22624_app1.docx]

**Multimedia Appendix 1: Confusion Matrix**

| Predicted Vs.  Actual | Yes | No |
| --- | --- | --- |
| Yes | 268 | 264 |
| No | 89 | 1568 |
