# Supplementary material for: Topics, Trends, and Sentiments of Tweets About the COVID-19 Pandemic: Temporal Infoveillance Study
Source: J Med Internet Res. 2020 Oct 23;22(10):e22624. doi: 10.2196/22624 (PMC7588259; doi:10.2196/22624)
Supplement: Multimedia Appendix 2 [file jmir_v22i10e22624_app2.docx]

**Multimedia Appendix 2: Themes, Topics and Associated Keywords**

| **Themes and Topics** | **Keywords** |
| --- | --- |
| 1. Source (Origin) |  |
| 1.1 Outbreak | chinese, wuhan, china, animal, bat, eat, meat, wet, market, outbreak, start |
| 1.2 Alternate Causes | bio, weapon, bioweapon, biowar, lab, conspiracy, cause |
| 2. Prevention |  |
| 2.1 Social Distancing | work, home, isolate, quarantine, outdoor, ban, social, distance, gather, avoid, feet |
| 2.2 Disinfecting and Cleanliness | Disinfect, alcohol, hand, hygiene, wash, water, sanitize, sanitizer, soap, clean |
| 3. Symptoms | exposure, mild, severe, respiratory, illness, fever, difficulty, symptom, sick, cough, shortness, breathe, breathing, chills, shaking, muscle, pain, headache, sore, taste, smell, flu, pressure, chest |
| 4. Spread and Growth |  |
| 4.1 Modes of transmission | air, tiny, droplets, sneeze, touch, mouth, nose, eyes, aerosol, transmit, airborne, asymptomatic, community, surface |
| 4.2 Spread of cases | spread, case, report, confirm, new, first, number, positive |
| 4.3 Hotspots and Location | hotspot, cruise, passenger, onboard, religious, cities, location, community, nursinghome |
| 4.4 Death Reports | deaths, die, kill, dead |
| 5.Treatment and Recovery |  |
| 5.1 Drugs and Vaccines | Med, medicine, vaccine, drugs, clinical, trials, chloroquine, hydroxychloroquine, drug, remendisivir, antimalarial |
| 5.2 Therapies | plasma, heat, therapy, inject, herd, immunity, blood |
| 5.3 Alternate methods | herb, plant, urine, dung, neem, lemon, Ayurveda, yoga, traditional, ancient, medicine |
| 5.4 Testing | test, kit, testing, rapid, swab |
| 6. Impact on Economy and Market |  |
| 6.1 Shortage of Products | shortage, empty, food, meat, scarce, grocery, available |
| 6.2 Panic Buying | panic, buy, sold, out, toiletpaper, toilet, paper, hoard, stock, lines, store, stores |
| 6.3 Stock Markets | stock, market, wallstreet, dow, shares, recession, economy, crash, collapse, depression, decline |
| 6.4 Employment | jobless, jobs, lost, unemployment, employed, layoffs, fire, downsize, interview |
| 6.5 Impact on Business | factory, industry, company, business, finance, shutdown, loss, loose, profit, impact, closure, close, open |
| 7. Impact on Healthcare Sector |  |
| 7.1 Impact on Hospital and Clinics | hospital, beds, clinics, clinicians, facility, ICU, intensive |
| 7.2 Health Policy | health, policy, ventilator, safety, guideline, triage, treatment |
| 7.3 Frontline Workers | Frontline, workers, nurse, doctor, physician, staff, enforcement, healthworker, healthemployee, nurses, socialworker |
| 8. Government Response |  |
| 8.1 Travel Restrictions | air, travel, border, cancel, close, flights, ban, transport, airline |
| 8.2 Financial Measures | refund, stimulus, package, regulatory, relief, payment, check, allocate, claim, pension, money |
| 8.3 Lockdown Regulations | stay, order, mandate, restriction, lockdown, announce, shut, shelter, signed, stayathome |
| 9. Political Impacts | politics, political, election, vote, elect, democrat, republican, party, congress, minister, president |
| 10. Racism | Chinese, asian, chinesevirus, racist, racism, anti, sentiment, fear, xenophobic |
