# Supplementary material for: Topics, Trends, and Sentiments of Tweets About the COVID-19 Pandemic: Temporal Infoveillance Study
Source: J Med Internet Res. 2020 Oct 23;22(10):e22624. doi: 10.2196/22624 (PMC7588259; doi:10.2196/22624)
Supplement: Multimedia Appendix 3 [file jmir_v22i10e22624_app3.docx]

## Multimedia Appendix 3

Figure S1 Trends in the proportions of positive, neutral, and negative tweets by theme. Color legend: green=positive, red=negative, and yellow=neutral.

| 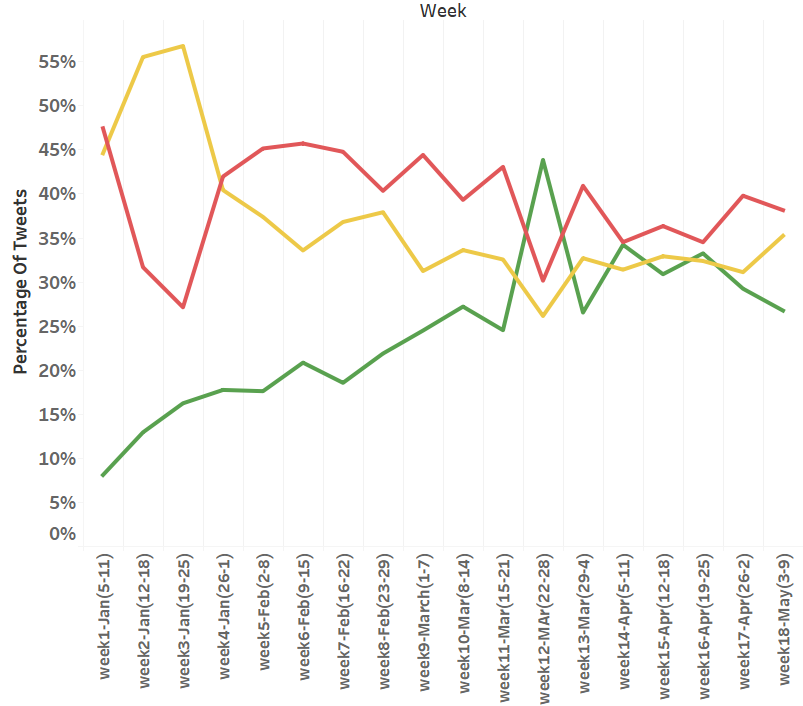  Theme: Source of the pandemic (n=966,372) | 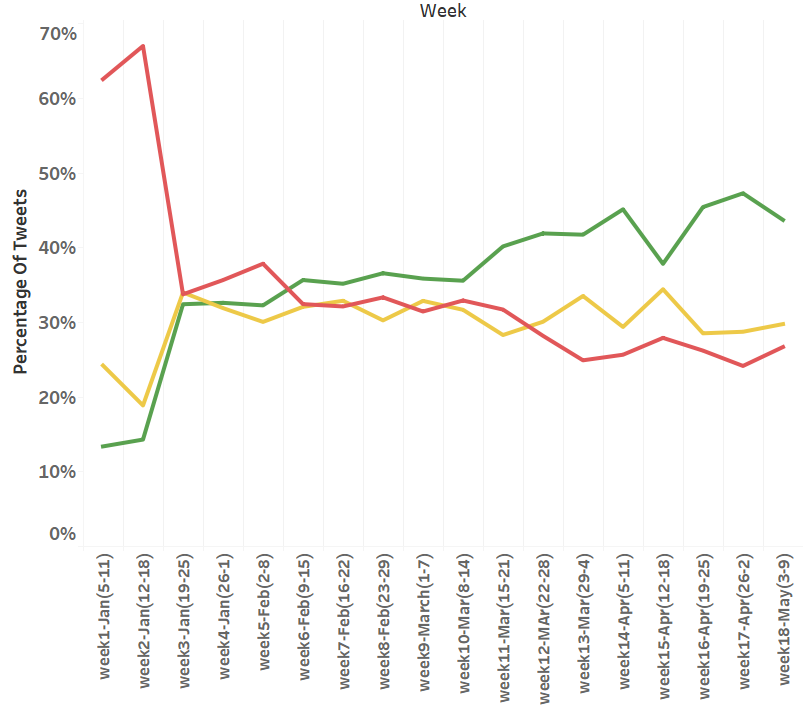  Theme: Prevention (n=1,076,840) |
| --- | --- |
| 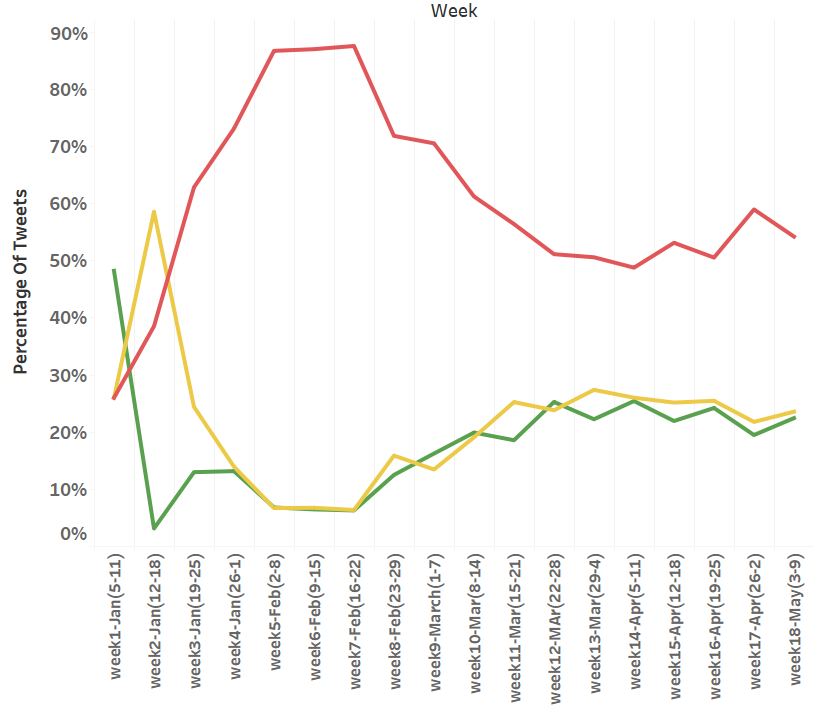  Theme: Symptoms (n=558,332) | 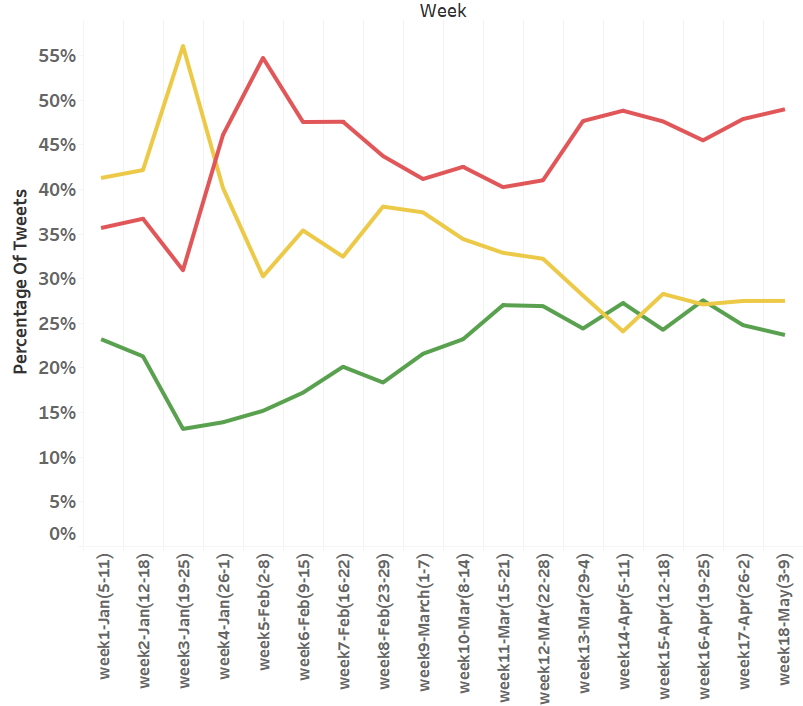Theme: Spread and increase in cases (n=2,154,065) |
| 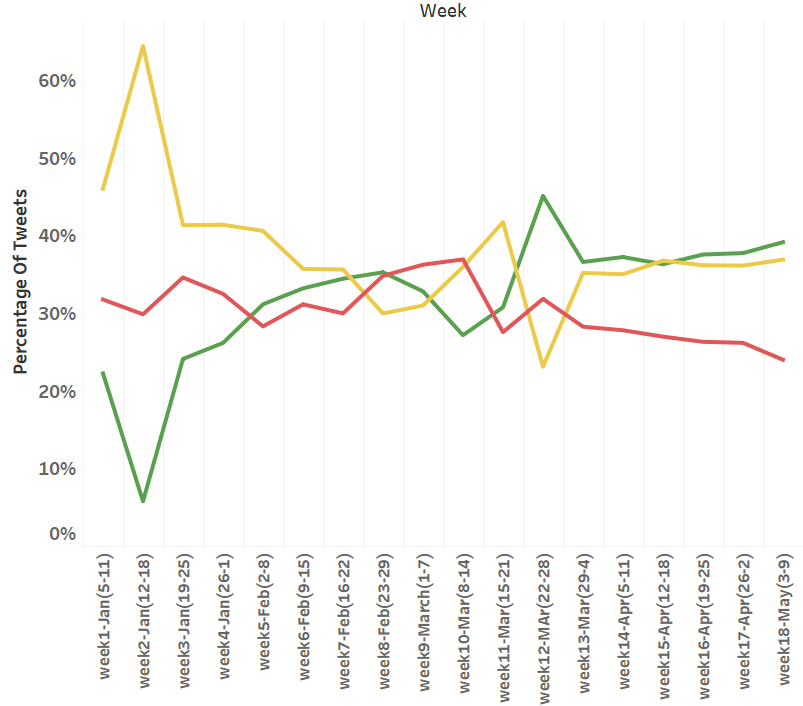  Topic: Treatment and recovery (n=1,831,339) | 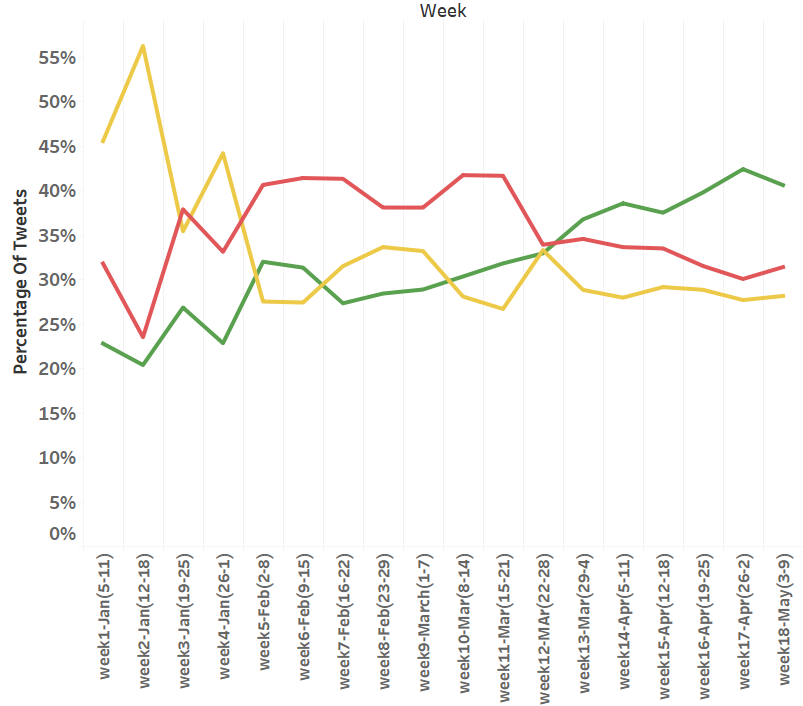  Topic: Impact on the economy and markets (n=2,858,316) |
| 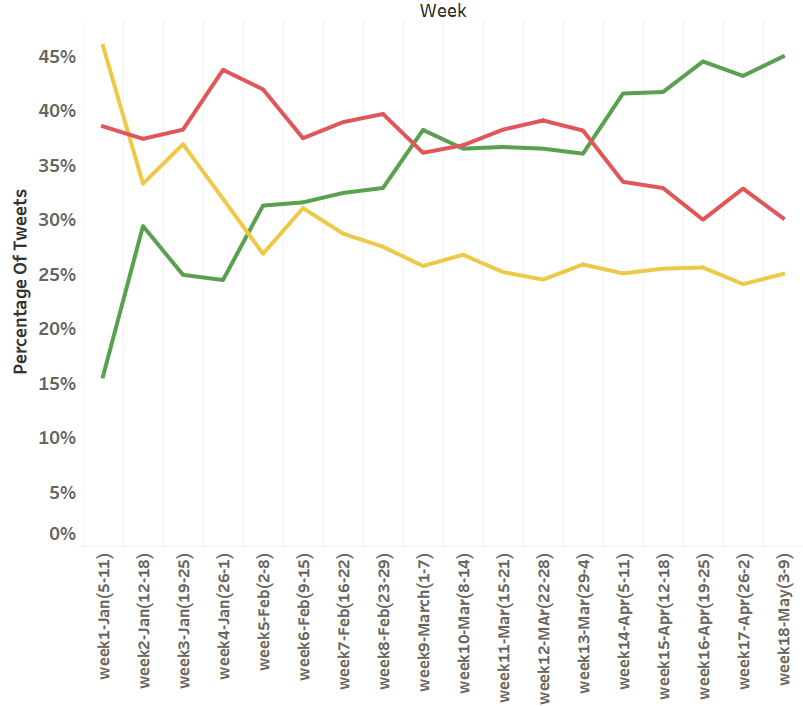  Topic: Impact on health care industry (n=1,588,499) | 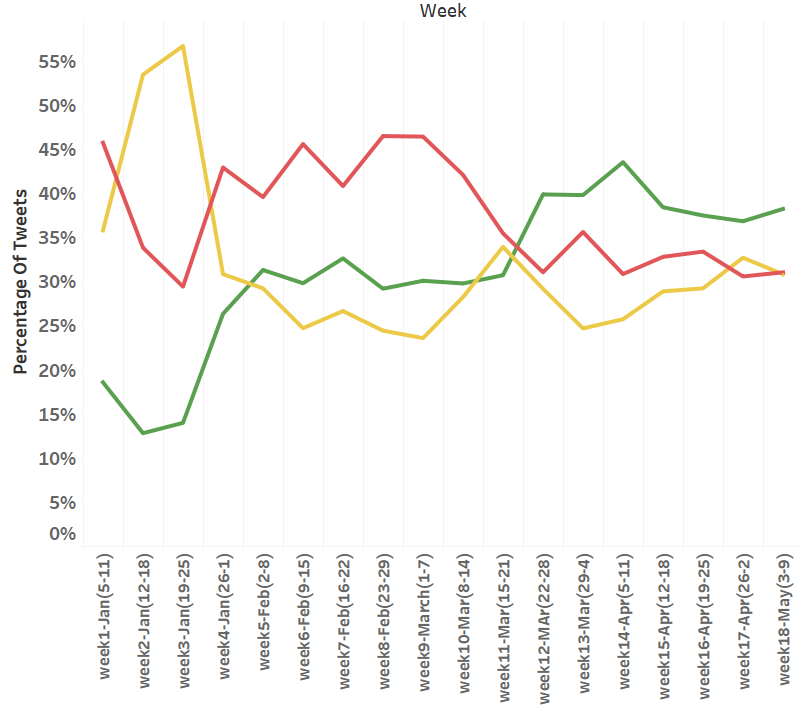  Topic: Government response (n=1,559,591) |
| 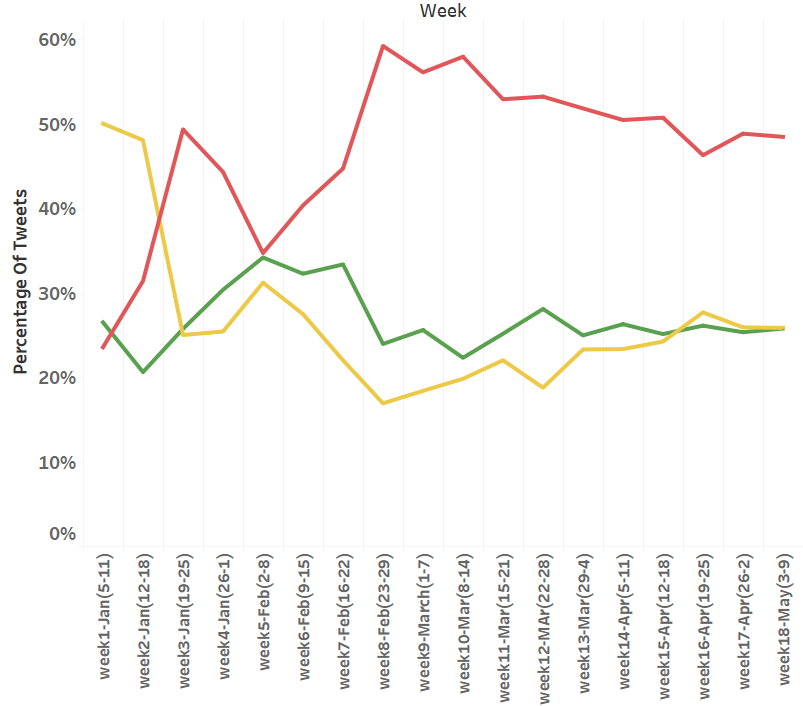  Theme: Impact on politics (n=767,486) | 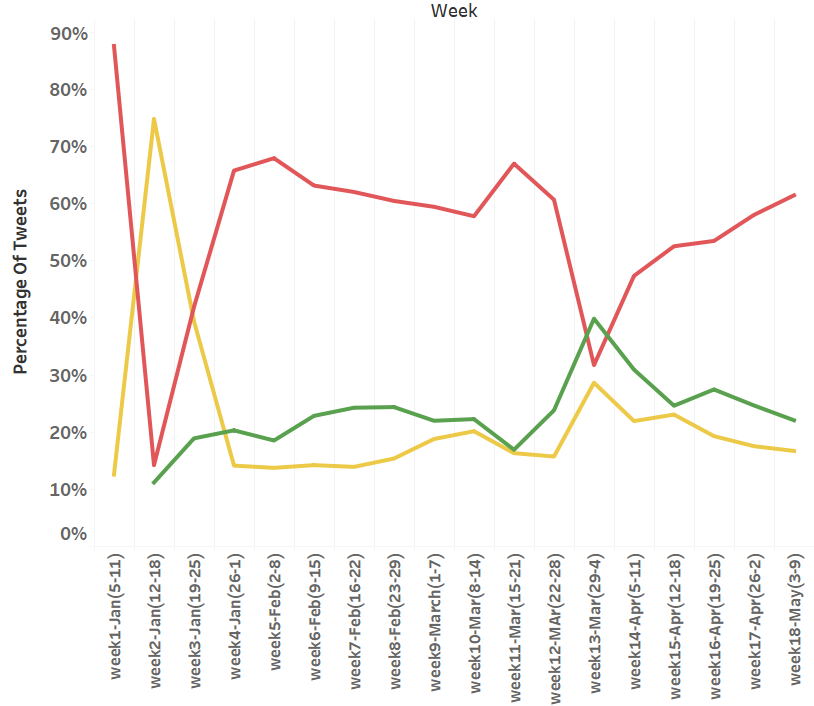  Theme: Racism (n=577,066) |

Figure S2. Sentiment score trends by theme.

| 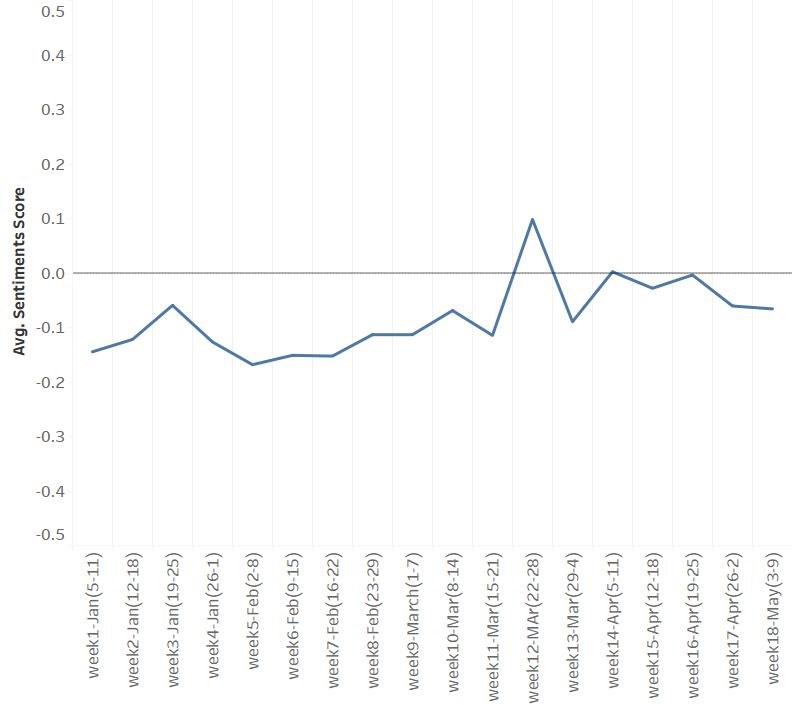  Theme: Source | 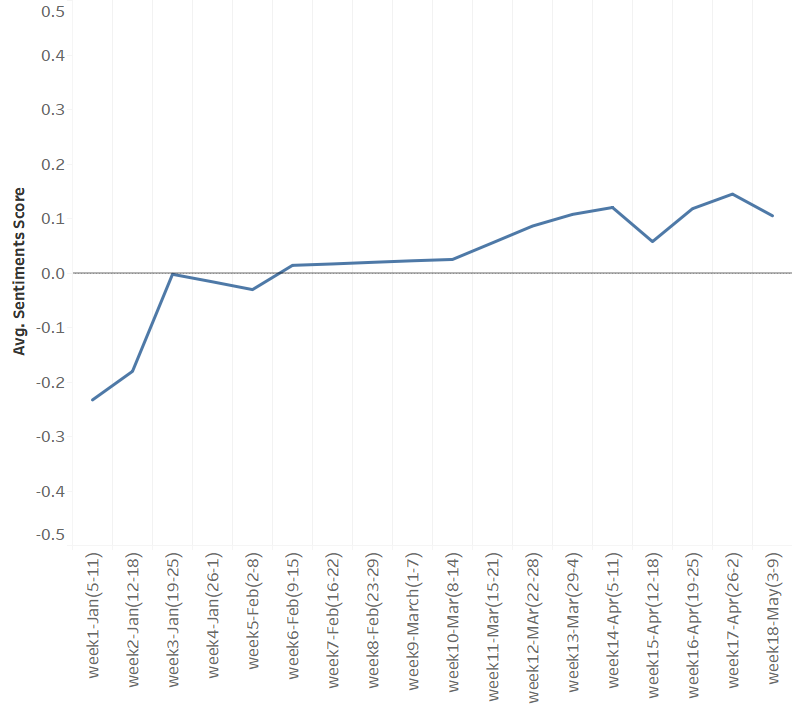  Theme: Prevention |
| --- | --- |
| 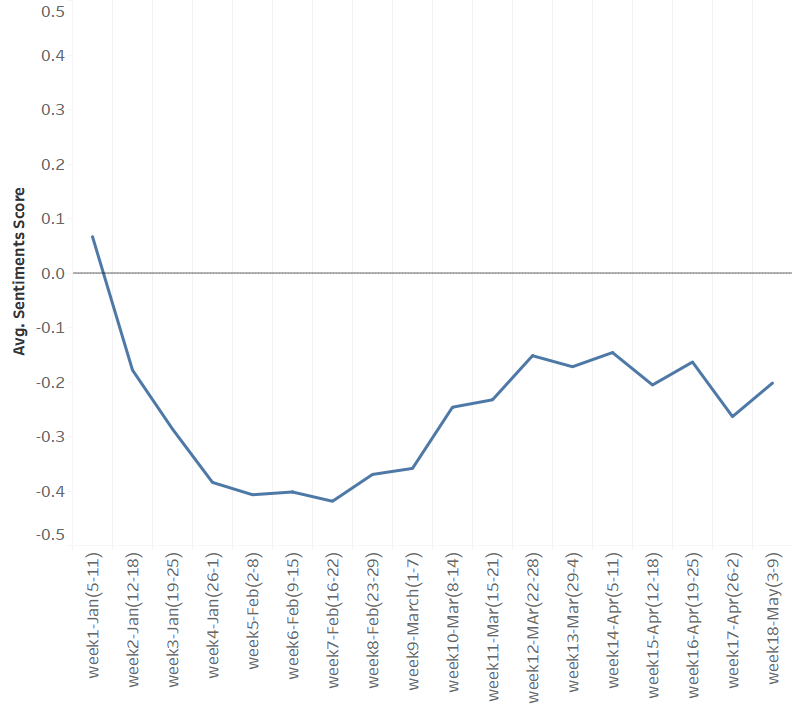  Theme: Symptoms | 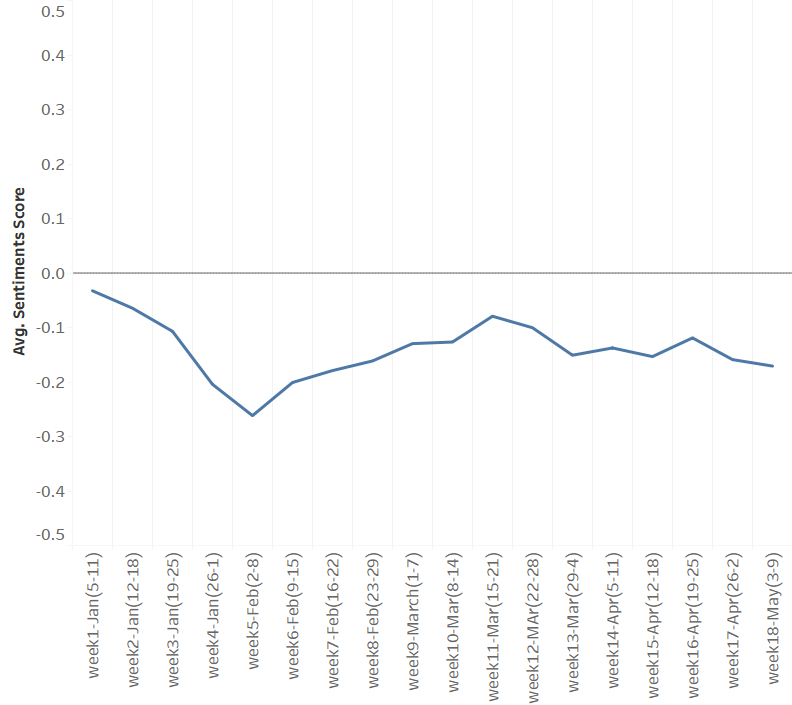  Theme: Spread and increase in cases |
| 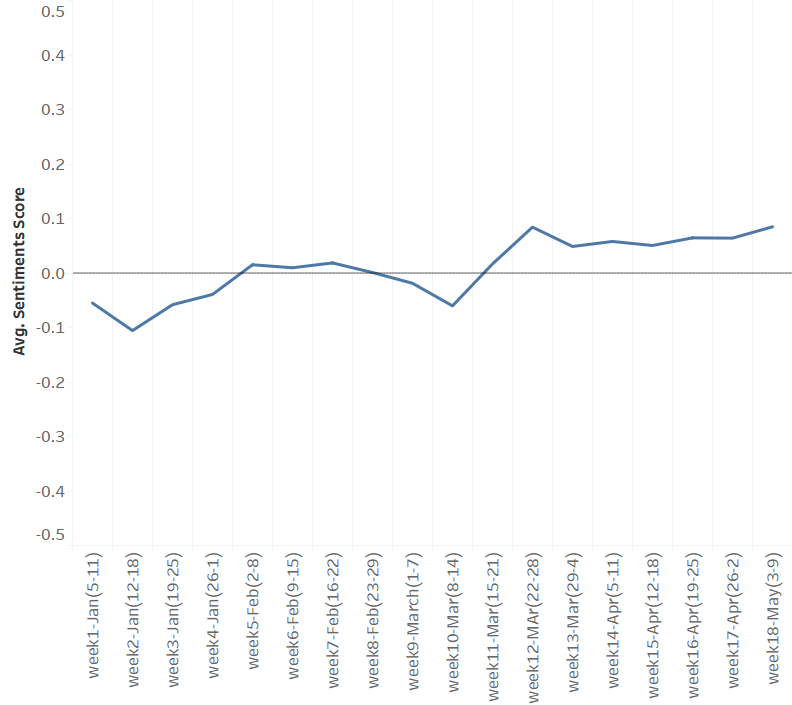  Theme: Treatment and recovery | 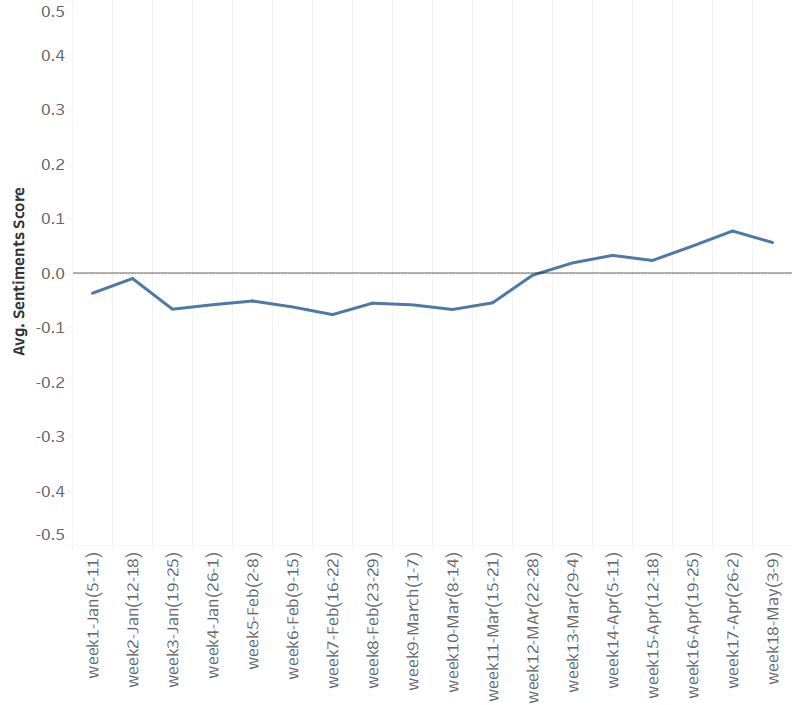  Theme: Impact on economy and markets |
| 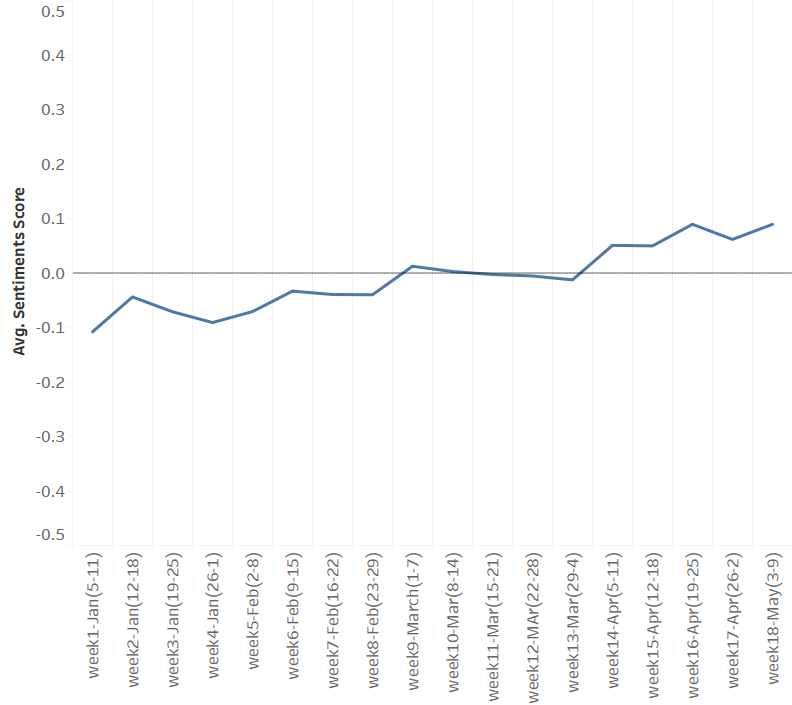  Theme: Impact on health care | 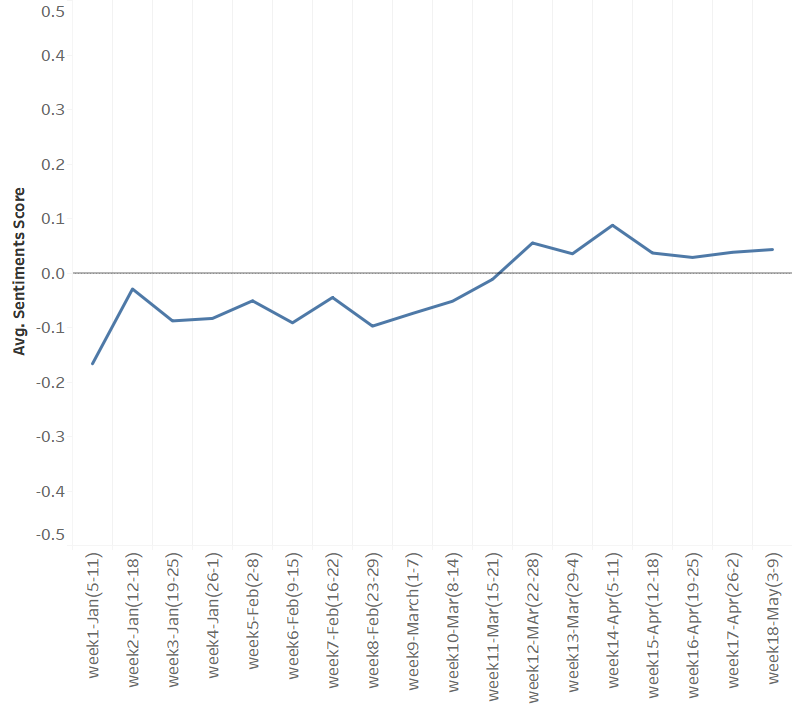  Theme: Government response |
| 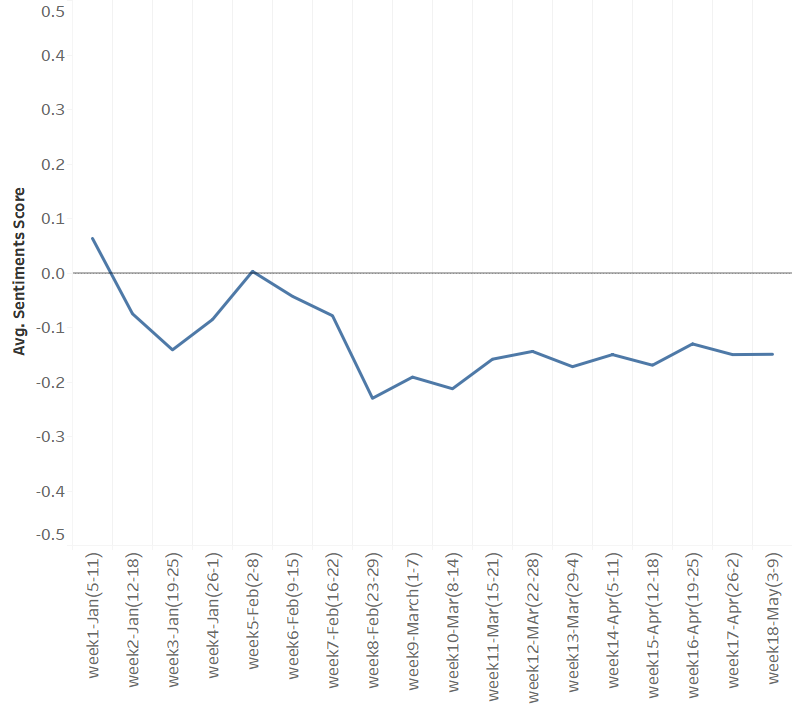Theme: Impact on politics | 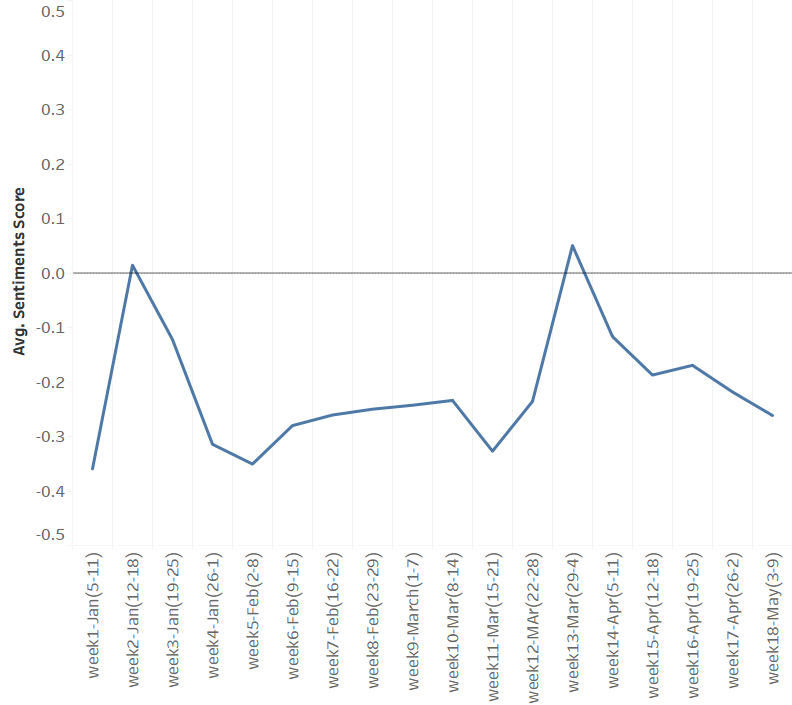  Theme: Racism |

Figure S3. Trends in sentiment scores by topic.

| 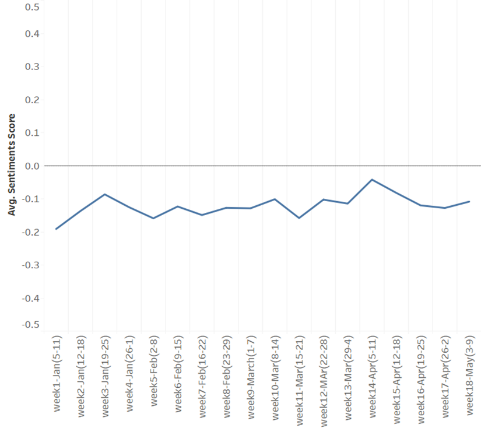  Topic: Outbreak (Theme: Source) | 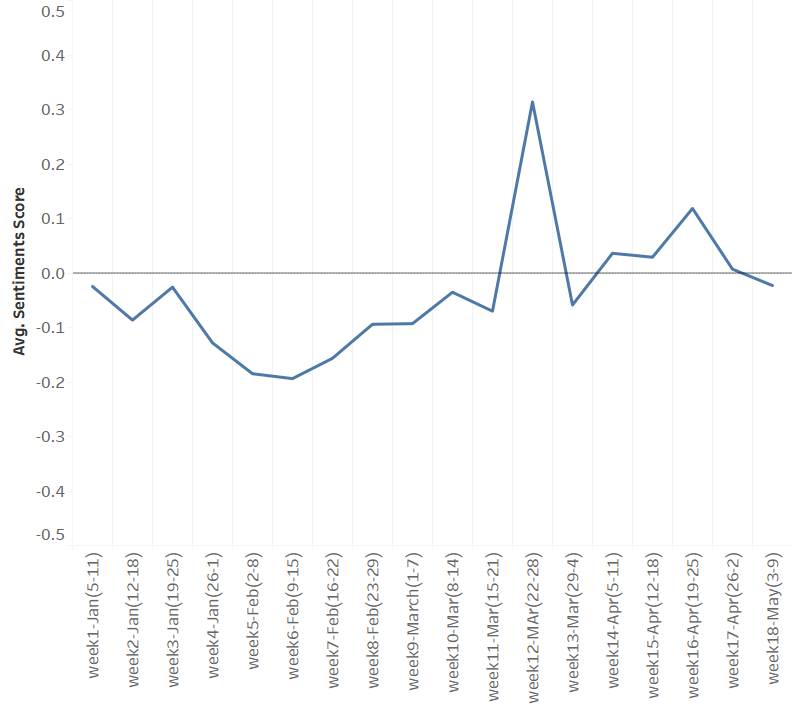  Topic: Alternative causes (Theme: Source) | 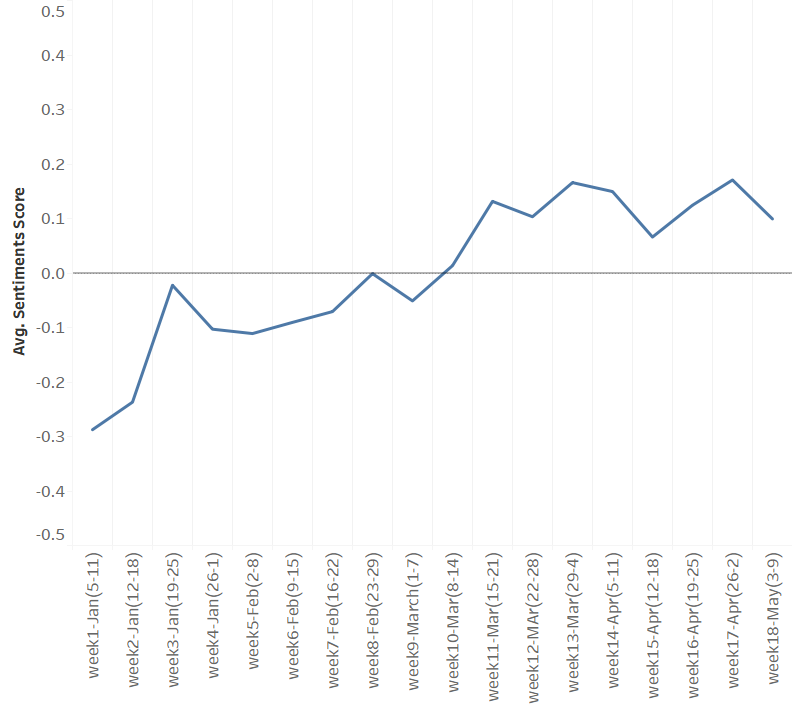  Topic: Social distancing (Theme: Prevention) | 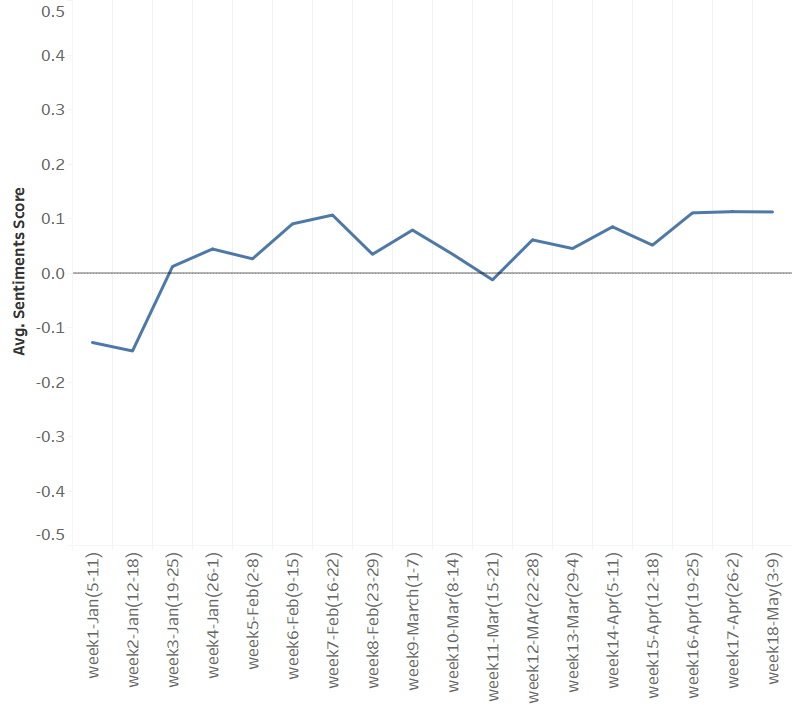  Topic: Disinfecting and cleanliness (Theme: Prevention) |
| --- | --- | --- | --- |
| 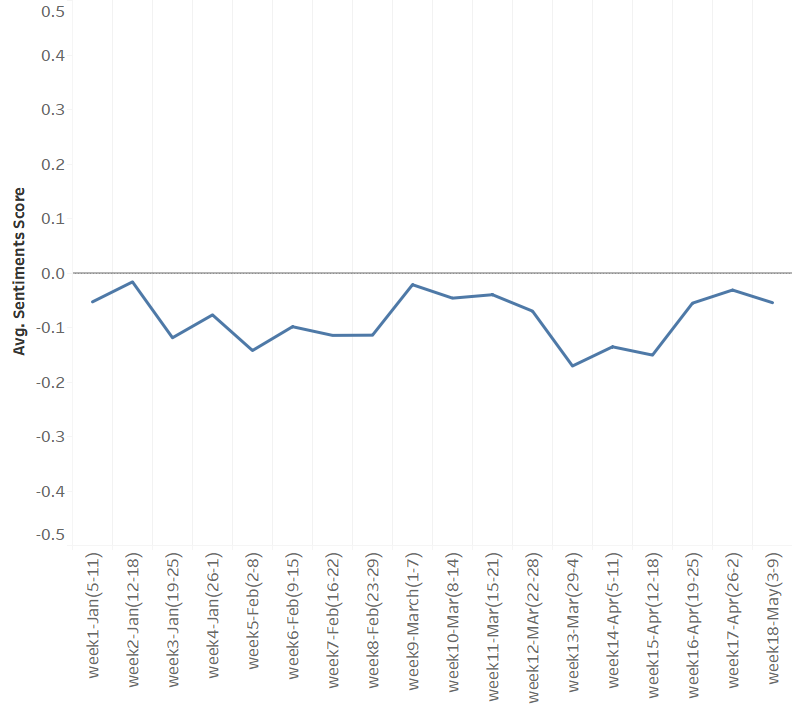  Topic: Modes of transmission (Theme: Spread and growth) | 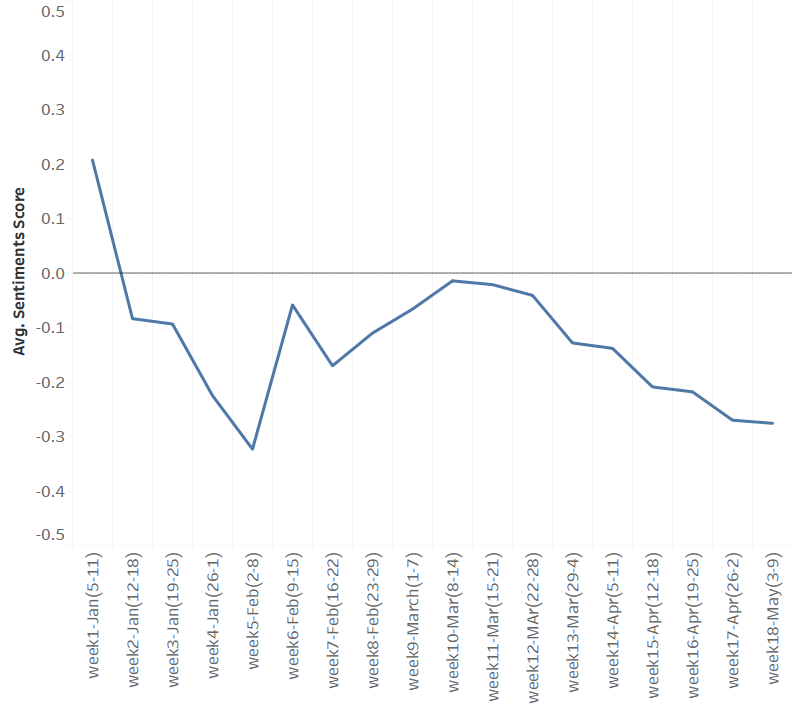  Topic: Spread of cases  (Theme: Spread and growth) | 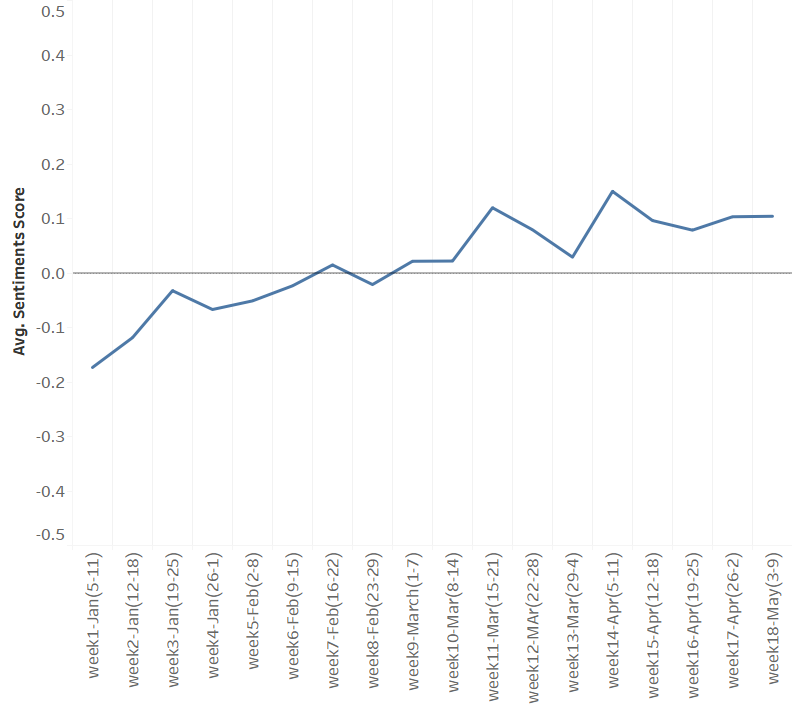  Topic: Hotspots  (Theme: Spread and growth) | 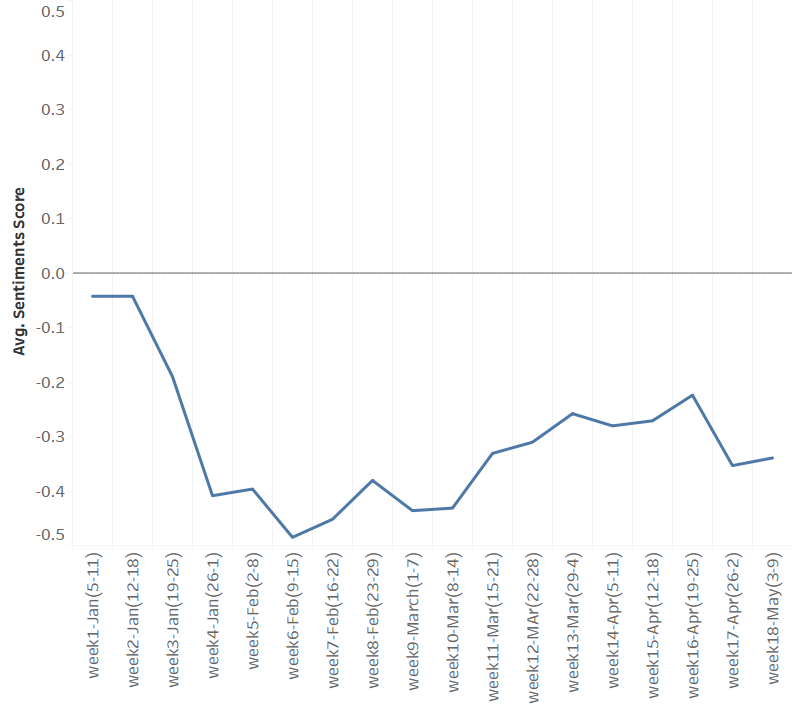  Topic: Deaths  (Theme: Spread and growth) |
| 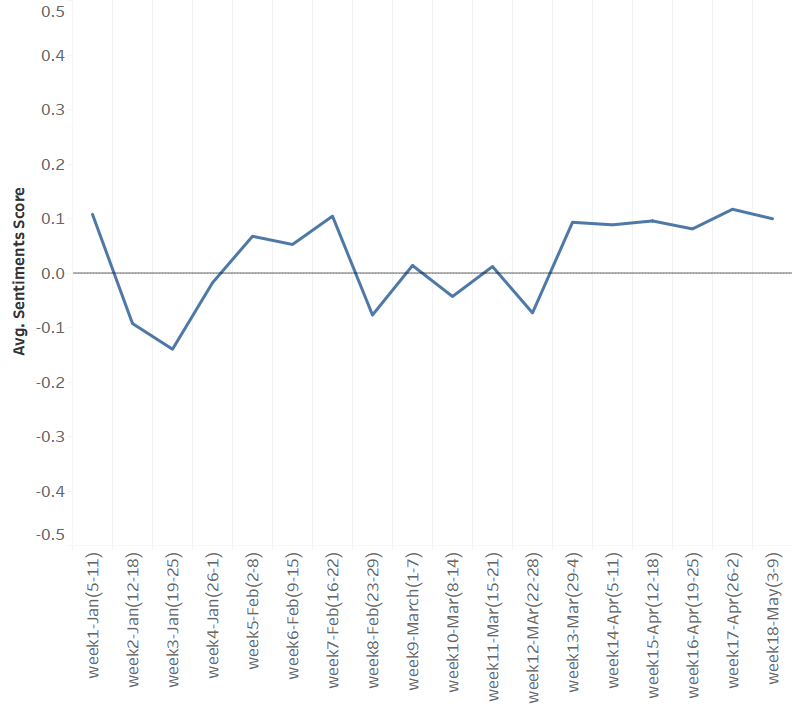  Topic: Drugs and vaccines (Theme: Treatment and recovery) | 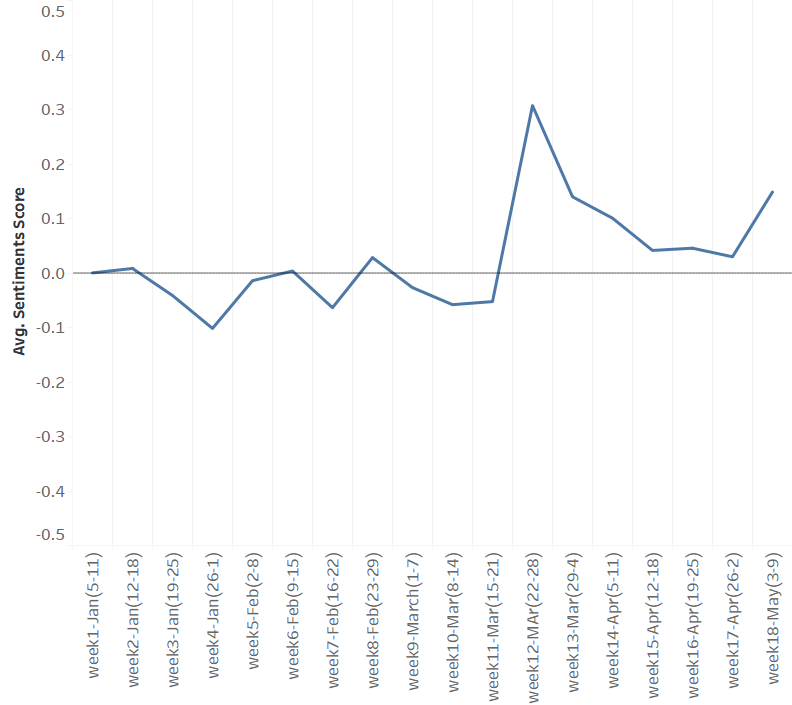  Topic: Therapies (Theme: Treatment and recovery) | 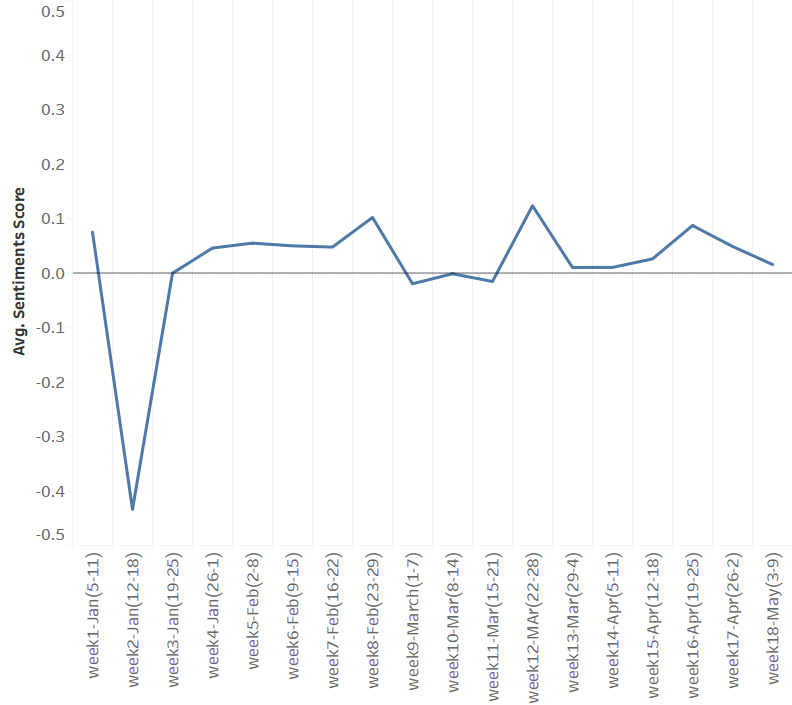  Topic: Alternative methods (Theme: Treatment and recovery) | 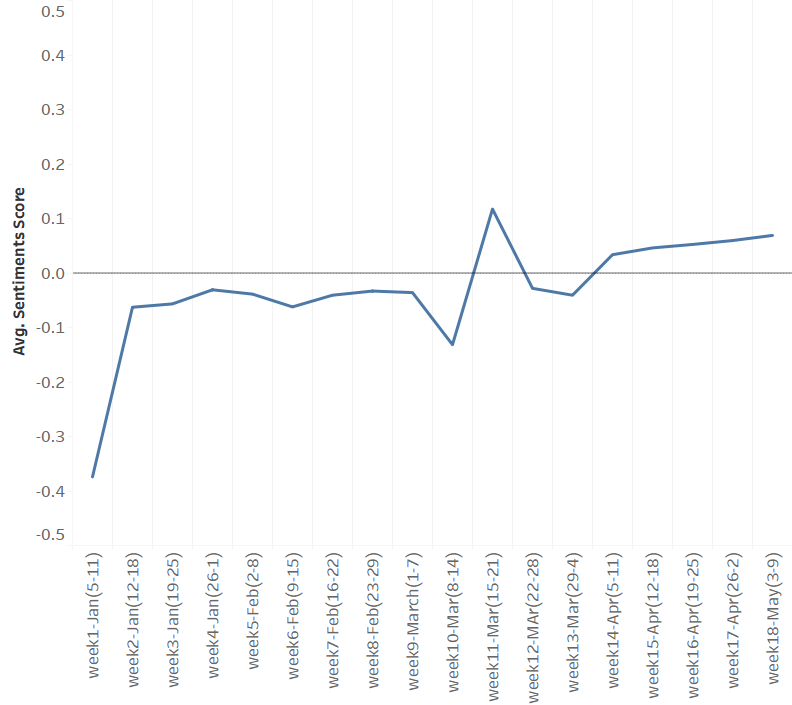  Topic: COVID-19 testing (Theme: Treatment and recovery) |
| 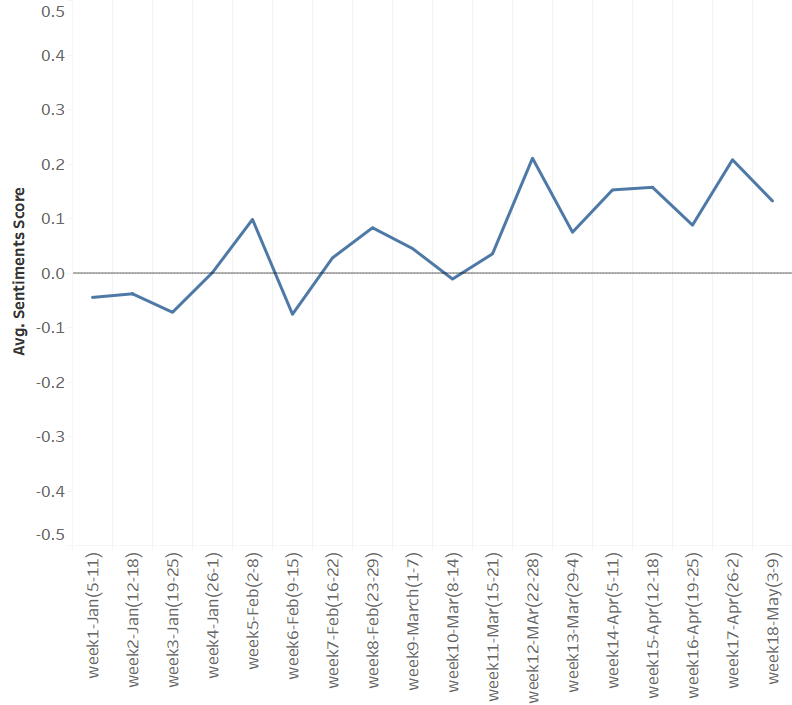  Topic: Shortages (Theme: Economy and markets) | 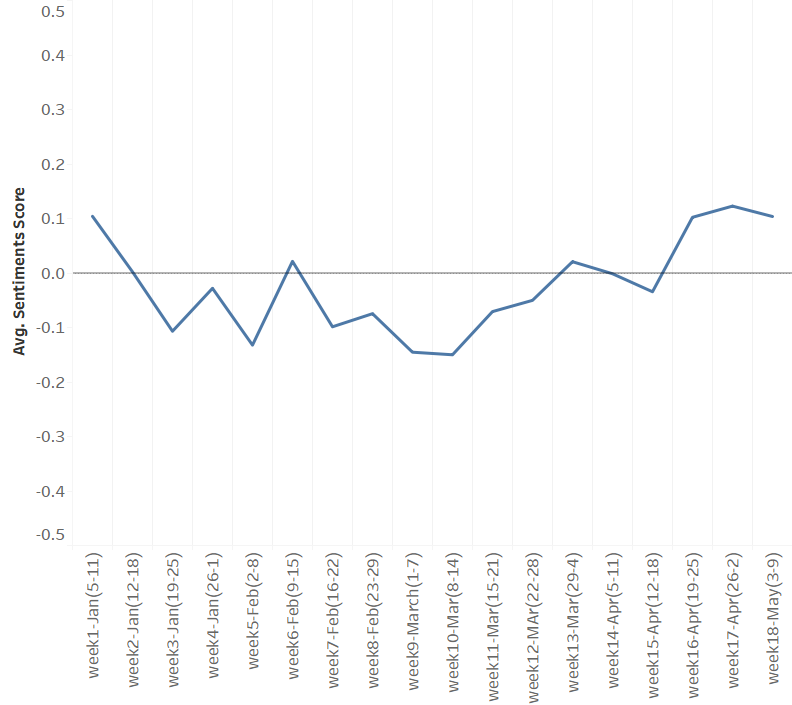  Topic: Panic buying (Theme: Economy and markets) | 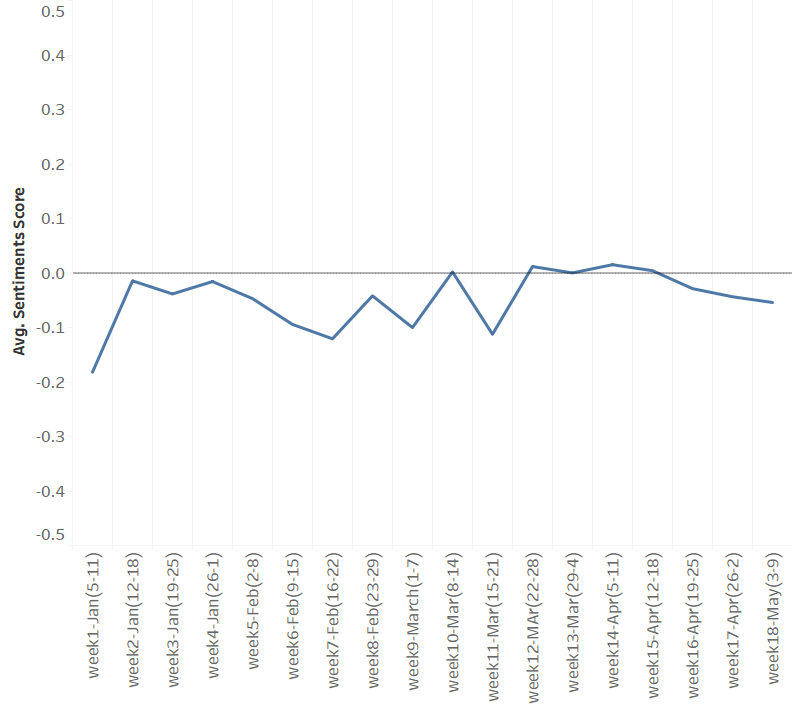  Topic: Employment (Theme: Economy and markets) | 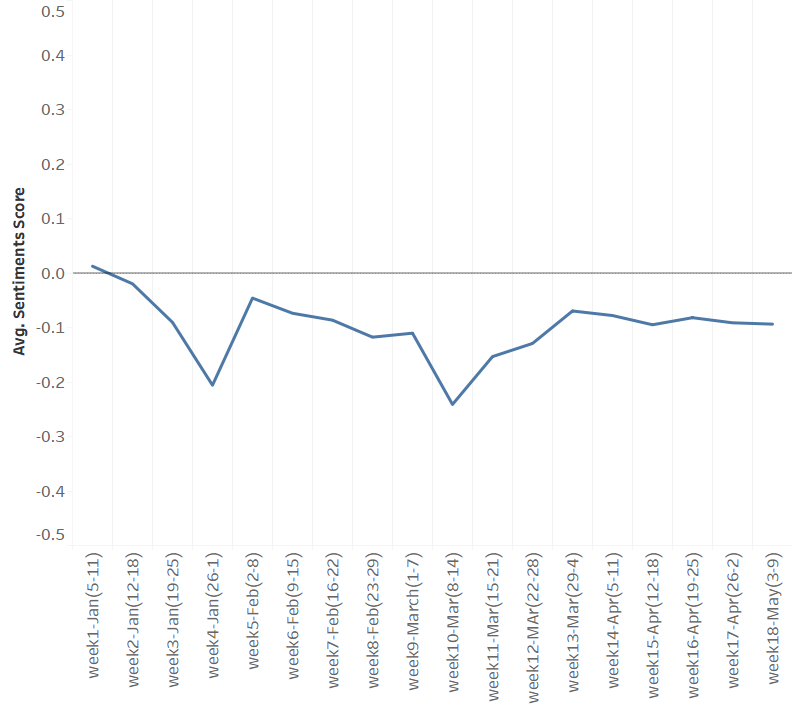  Topic: Stock markets (Theme: Economy and markets) |
| 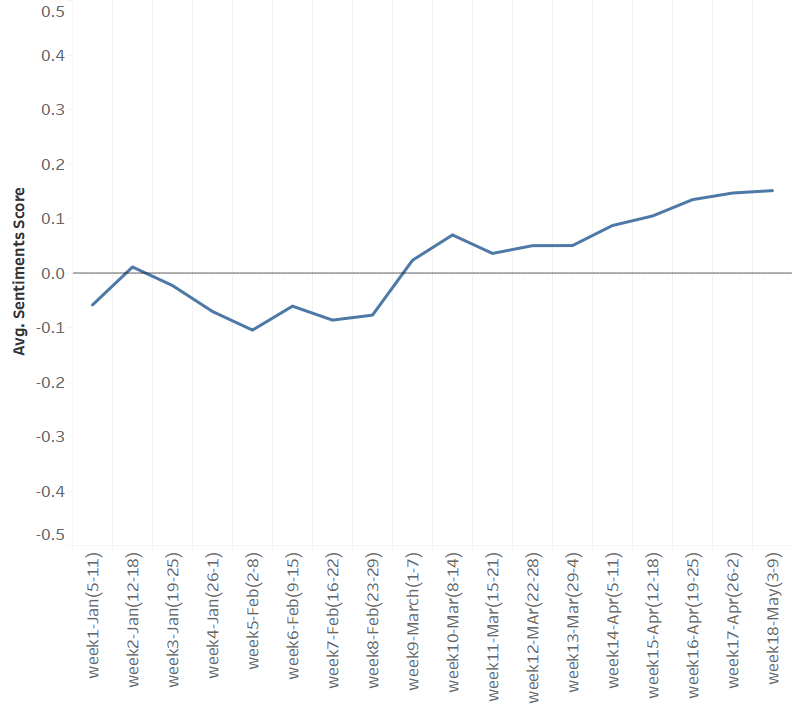  Topic: Businesses (Theme: Economy and markets) | 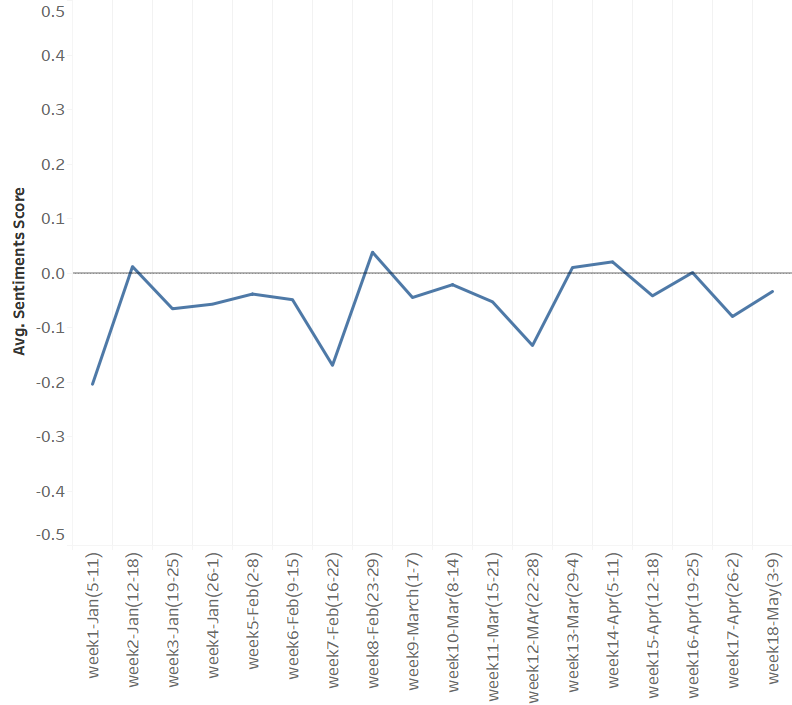  Topic: Hospitals and clinics (Theme: Health care) | 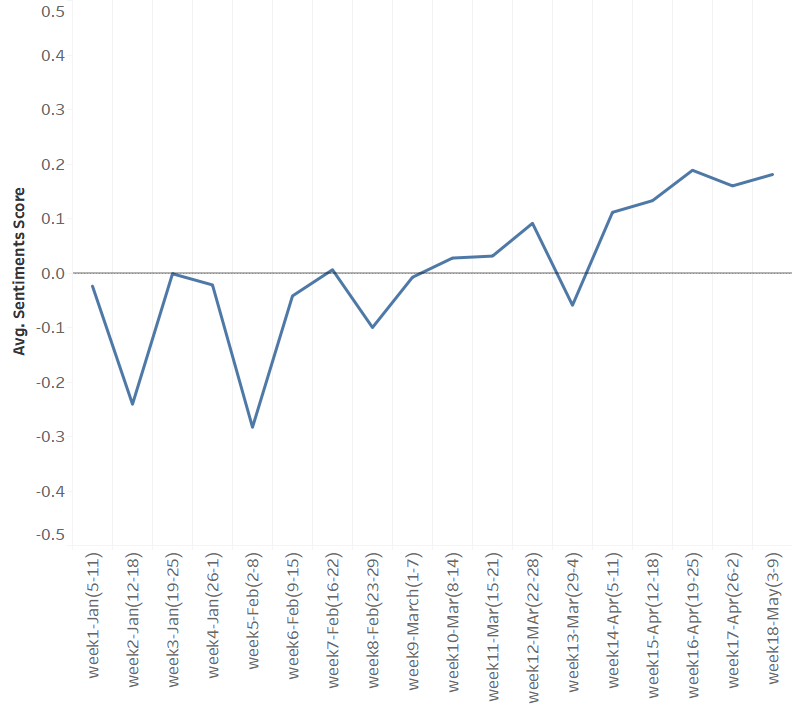  Topic: Frontline workers (Theme: Health care) | 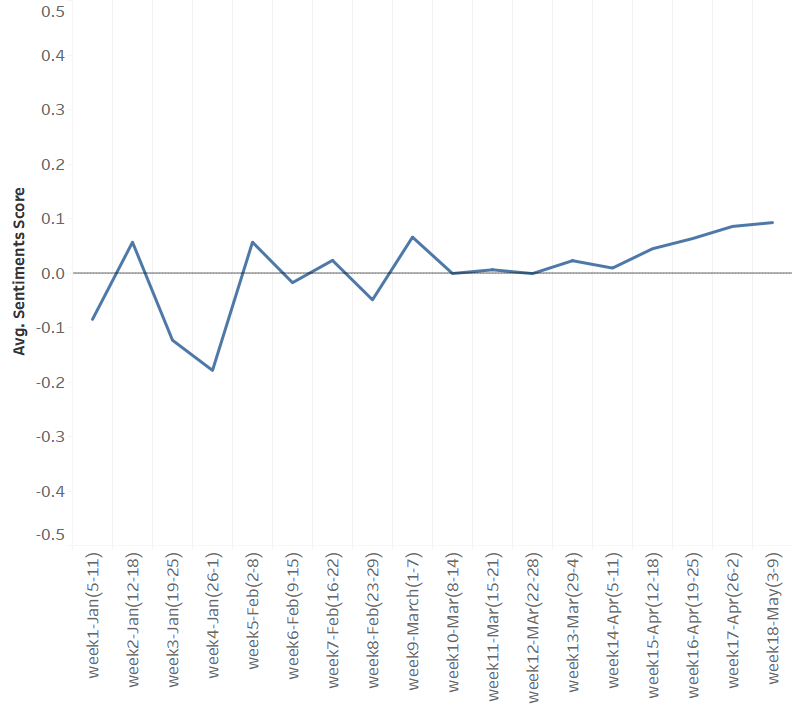  Topic: Health policy (Theme: Health care) |
| 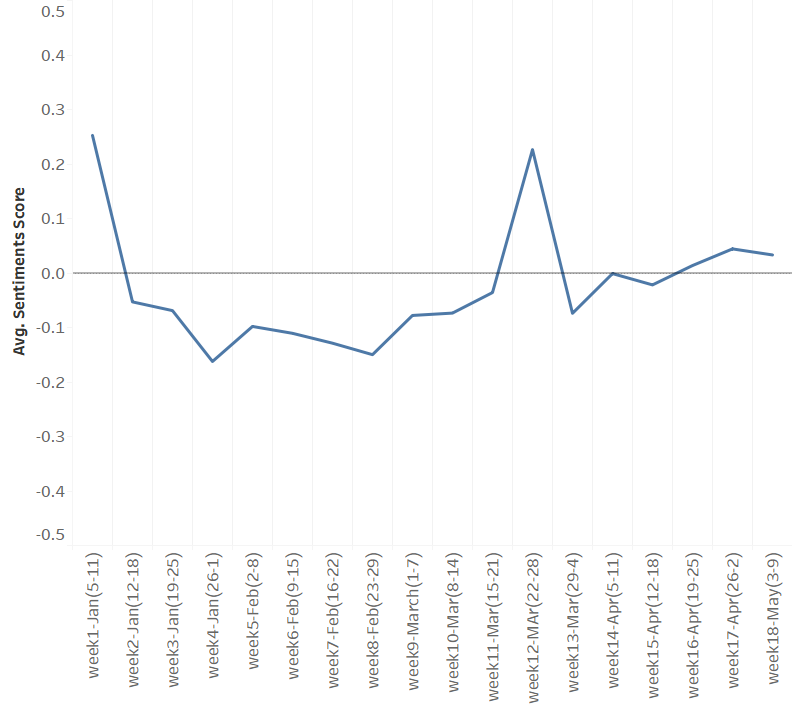  Topic: Travel restrictions (Theme: Government response) | 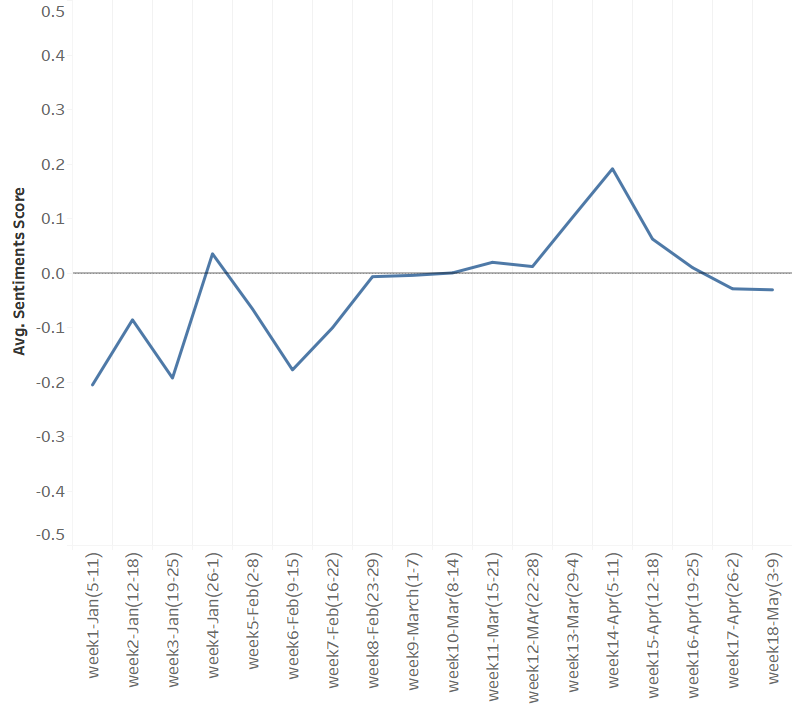  Topic: Lockdown regulations (Theme: Government response) | 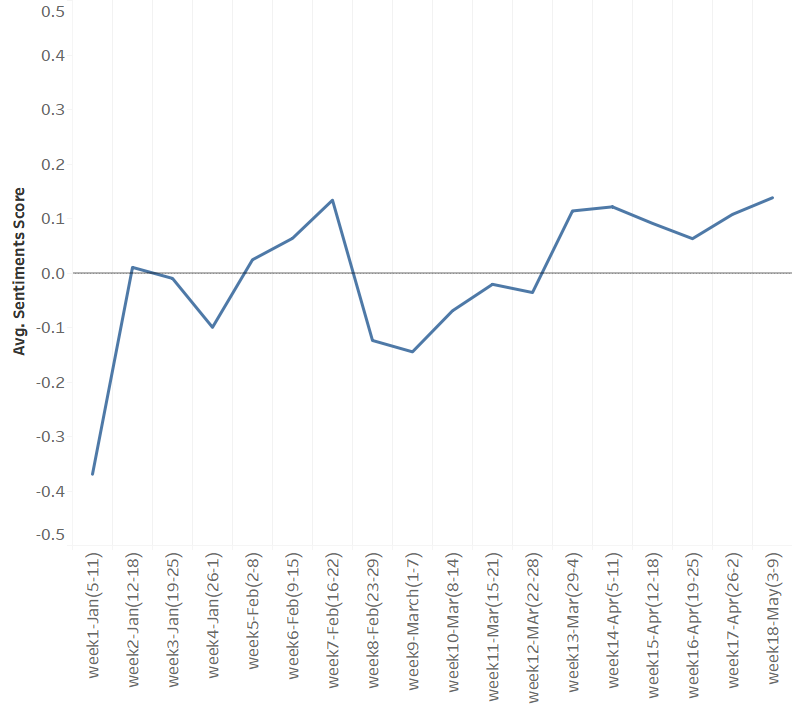  Topic: Financial measures (Theme: Government response) |  |
