# Supplementary material for: Topics, Trends, and Sentiments of Tweets About the COVID-19 Pandemic: Temporal Infoveillance Study
Source: J Med Internet Res. 2020 Oct 23;22(10):e22624. doi: 10.2196/22624 (PMC7588259; doi:10.2196/22624)
Supplement: Multimedia Appendix 4 [file jmir_v22i10e22624_app4.docx]

**Multimedia Appendix 4**

**Illustrative tweets for Themes and Topics on COVID-19 discussion on Twitter**

| **Topic** | **Illustrative Tweets** |
| --- | --- |
| Outbreak | Ground zero for the Corvid -19 was the Wuhan meat market where many species of wildlife were sold.  The prime suspect for human transmission of Wuhan Coronavirus is Pangolin in Wuhan’s meat market. China has banned consumption of wildlife animals after the outbreak.  it was reported virus started at Wuhan MEAT market.. bat->pig->pig handler->customer.  If the Chinese government has nothing to hide in how it responded to the coroanvirus outbreak in Wuhan, why is it opposing an independent international investigation?  China Unleashes a Virus to the whole world and later blame Africans for it. This is unacceptable and we won't take it anymore. Wuhan is the source of Coronavirus and not Africa |
| Symptoms | I might have coronavirus, so that's fun. Persistent low-grade fever, light dry cough, light shortness of breath, fatigue, and I've mostly lost my sense of taste. ER (via phone) says they can't test me. Test shortage is so dire that tests are reserved for most urgent cases only.  Some personal COVID news. Yesterday after feeling shortness of breath, having a dry cough and frankly just not feeling well, I called my doctor who advised I get tested for COVID. You guessed it, I tested positive for COVID.  My symptoms started on Mar 19 with a dry, grating cough - this lasted for two weeks. For a few days prior to this I felt lethargic but ok.  So the inevitable has happened, developed a persistent, dry cough overnight, gonna need to self isolate.  Sooooo I am getting tested for COVID-19. Have had a slight dry cough for several days, no other symptoms, no travel or contact history, had a call with GP this morning and they are sending me the at-home test kit this afternoon |
| Alternate Causes | This virus is certainly God’s call to humanity to wake up and recognise him before it is too late.  Wow. Earth is recovering, Air pollution is slowing down, Water pollution is clearing up, Natural wildlife is returning home, Coronavirus is earth’s vaccine. We’re the virus”  This planet will surely heal, in the most magical ways. I can feel the vibrations coming on.  Meat Eaters will be punished by God and put in hell. Today the curse (in the form of Corona Virus) of hurting animals is being faced by China People who had committed many sins by eating animals.  This is not natural virus. This is biowar. Lab created virus.  It might have been a cover-up of the real source of the Pandemic - an accidental leak of a prototype BioWar virus from Fort Detrick (MD).  Add a bioweapon virus stolen from Canada by the Chinese that leaks in a high-level government facility 20 miles from Wuhan  Bioweapons created in labs like the one in wuhan (less than a mile from the patient one in good market) can carry nanoparticles that can get even more scitivster when exposed to microwave radiation (5G)  That virus is a BIOWEAPON which China was gonna use against us because all the sanctions the U.S. has put on China, but things got out of hands and shit backfired, so now China has to assume responsibility for not only the economy worldwide but for all deaths!! THAT'S THE TRUTH.  According to the chinese report the Corvid-19 originated from the visiting 300 US military to wuhan China for the Military World Games. They infected the local population. |
| Social Distancing | Can’t believe the amount of people acting like everything is back to normal, seen zero social distancing on my snapchat and insta today  I’m really disturbed how people think their “social freedom” is more important than the lives and health of others. No social distancing! How idiotic and selfish can one be to really wanna risk a resurgence of the Covid spreading. I don’t get it.  this is today's the pics of kandivali mathuradas market. I can't see any social distancing over here. Market is full of people, who don't care for their life and others.  Went to the grocery store this morning a bit self conscious wearing a facemask (made of recycled tees, that were made of recycled plastic bottles thank to allmadeapparel). 1 of 4 people in there were wearing masks, practicing social distancing, and overall respectful of space.  Kindly stay at home . Wash your hands. Practice social distancing.  the whole world needs to come together and work together on a common goal a worldwide quarantine and social distancing is the only way to beat this coronavirus  Ran two miles even when I didn’t want to! Made excuses all day! Get out there and do it! But practice social distancing, let’s flatten this curve!  We know the dangers of asymptomatic COVID-19 carriers spreading the virus. That's why we social distance.  Social distancing paradox. We social distance because we know we have to, and when it succeeds and nothing bad happens, we feel like we all did it for nothing because nothing bad happened. It’s logical I suppose in a way, but eye on the prize people. We aren’t even done yet! |
| Disinfecting and Cleanliness | Idk bout y’all but I lowkey can’t breathe in these face mask  I see a lot of people without face masks and as cnn has been reporting for past three months without social distancing and masks we all die  Masks are utterly useless against virus particles  Evidence for hand-washing effectiveness for COVID-19 is basically non-existent AFAICT.  My friend src2 challenged me to do the #SafeHandsChallenge  ! It’s important to make sure you’re using proper hand washing hygiene to help contain the spread of COVID-19.  my groceries are being delivered today. The washing, wiping, and disinfecting of food products will begin sometime mid to late morning.  Hand washing and no handshakes should be permanent even if Covid eradicated.  Wearing a face mask is one of the most powerful things we can do to protect others. |
| Modes of transmission | Why does the WHO say that surface transfer is a major vector for transmission of COVID-19 and the CDC says it's not? Why does WHO emphasize you can catch it from an infected but asymptomatic person exhaling and microscopic droplets transferring, and the CDC not?  i'm just wondering what possibility there is of corona virus being spread through air conditioning?  If you ever get COVID, it’s likely coming from a human droplet, instead of shoes or clothes or boxes or ATMs or groceries  when it is noisy, people raise their voices to be heard, which facilitates droplet transmission. I'm not suggesting that, like, noise gives you covid.  Generally [#Covid_19](https://twitter.com/hashtag/Covid_19?src=hashtag_click) survives on any surface for 9to12hrs  For months experts said the primary transmission method for COVID-19 was "touching your face from a contaminated surface." We now know it's almost ENTIRELY transmitted via the air. |
| Spread of cases | Covid cases in US: Mar.8: 558. April 8: 432,132. May 8: 1, 283, 908. If a mask is good enough for Batman, a mask is good enough for me.  Western Cape Covid-19 cases spreading rapidly in the past 10 days. Time for an intervention and assistance by the DoH?  COVID-19 cases have skyrocketed in TX prisons, w/ virus infecting & killing incarcerated people & staff, spreading into nearby communities through workers who travel back & forth each day.  Singapore should be a warning to us. They had COVID under excellent control. Now it’s out of control: 728 new cases yesterday  We currently have 1,346,332 confirmed cases and 80,004 deaths from COVID-19 in 70 days. 80,004 deaths is more than 27 attacks on 9/11. Averaging 1,962 COVID deaths per day since April 1, that translates into another 9/11 attack every 1.52 days.  128,940 tests taken, numbers continue to rise in Arizona 10,960 COVID19 cases – my Mom is one of them! 532 COVID19 related deaths 1 pediatric death on May 7 67 deaths – highest in one day on May 8 |
| Hotspots | 11th different hospital visit in LA today LA is a Corona Virus Hot Spot... All Empty....Scary  It’s not good here. COVID19, hotspot, grandisland, Nebraska  folks -- Tippecanoe County may be a hidden hotspot -- keep staying home!!!!  In Belarus, churches and monasteries became a hotspot of COVID19 infection, especially due to the recent Easter celebrations. Expect more cases in the coming weeks  in the next two weeks GA is about to be a new hotspot |
| Deaths | as of saturday after noon: ny: 59,513 cases and 965 deaths nj: 11,124 cases and 140 deaths ct: 1,291 cases and 27 deaths new york and new jersey have the most confirmed cases in the U.S  The UK announced nearly 4000 extra COVID19 deaths and is about to become the 2nd worst affected country in the world.  More than 5,000 Brazilians have lost their lives to the coronavirus.  7 COVID 19 related deaths in Nigeria in the last 24 hours. Hmmm.  Covid is no joke. My ex MIL died yesterday, after she caught it in hospital when in for something else  if we don’t take further actions at slowing the COVID-19 outbreak, we could see the number cases to be in the millions and the number of deaths to be over 200,000 within a few weeks. |
| Drugs and Vaccines | My friend a 30-y-o NYC public school teacher—went on a ventilator on Sunday after catching COVID-19. Her family is desperate for a potentially life-saving treatment: the antiviral drug remdesivir.  Today, the world gets the first real sign that a new medicine, remdesivir from Gilead Sciences, will help the world emerge from the Covid-19 pandemic.  Remdesivir is effective in mitigating COVID-19 symptoms if taken EARLY, ideally pre-hospitalization  Hydroxychloroquine protocol: effective, cheap and can be produced in many laboratories.  HCQ functions as both a cure and a vaccine.  There is no known medicine for COVID-19 yet. Different countries are trying different treatments e.g. HIV+Tamiflu+Chloroquine in India, Chloroquine+zinc in SKorea etc.  China found that using Sputum sucking machine was more effective than ventilator in many cases |
| Therapies | The effects of coronavirus are scary for many families, but this treatment of using antibodies from recovered patients could save lives. My husband John’s story is like so many & I’m so proud that he is helping others by donating his plasma.  Plasma donation round 2 ! Last time it helped get a patient out of ICU , Note from my Doctor “hoping all recovered covid patients come out and donate their blood , u may be able to help someone  If plasma therapy has shown beneficial results, then everyone who recovers from corona has a duty to donate plasma  plasma from recovered coronavirus patients would be expected to contain helpful antibodies and looks encouraging  We desperately need a treatment for those severely suffering with Coronavirus. Blood plasma could be the answer. The NHS is asking patients who have recovered from Coronavirus to donate blood for trials - visit NHS site to help.  The FDA said Plasma from recovered patients “may be effective against the infection”. You take antibodies from the blood plasma of a recovered patient & inject it into a seriously ill Coronavirus patient. FDA approved testing. It’s not a cure but a help to recover |
| Alternate methods | China is THE most Successful Country against COVID, they treated Patients with Chinese Herbal Medicine, in Gujarat State India, at 1 Quarantine Center many patients chose Ayurveda Herbal Medicine  herbs like neem and tulsi are so potent that can't imagine them not being useful vs Covid-19. think Ayurveda can come up with a cure.  For COVID19 there is no proper medicine available in allopathy. Ayurveda is giving results even in those disorders where allopathy is not effective.  My Chinese supplier claimed this to be their local herb they used in their hospitals to cure coronavirus. That after 10days, the patients will be fine.  Chinese medicine has made a great contribution to fighting COVID-19 in China. About 70,000 COVID-19 patients received Chinese medicine. The pandemic was well controlled within 3 months  Good news! Homeopathy is 100% as effective treating COVID-19 as it is treating literally anything else. |
| COVID-19 testing | I didn’t die from it, but I definitely had it and was unable to be tested due to the shortage of tests in NY  Many places are not retesting people for coronavirus due to test shortage. Its annoying  Ya don’t realise how scary coronavirus is until you test positive your self, I got my results back today And had the worst panick attack. Not showing the most serious side to it but still not well. Self isolation in my room for me to get better.  They have expanded testing criteria so anyone with even mildest symptoms can get one. There is no shortage of tests right now.  We got our COVID tests at the LA County drive-thru testing facilities! It was super fast and easy, and they only require you to swab your mouth, not your nose.  We got tested today. Easy as could be, no waiting, felt really safe, cheek swabs  In lots of states, it’s very easy to get tested now, even if you’re asymptomatic. |
| Shortage | COVID-19 is costing people their lives!!!, jobs, homes, & now meat processors announced food shortage warnings. this shit is getting worse by the day.  it's really jarring and upsetting to go grocery shopping these days and see so many empty shelves.  talked to my friends from Cuba today & they are suffering a food shortage in quarantine due to the US blockade.  I don't understand why people are freaking out about a meat shortage. I was vegetarian for 7 years, vegan an additional 2 years.. I know how to eat well without meat.  So, now with this self-imposed meat shortage, will we now be able to find toilet paper?  With the help of local authorities, my family reached out to 7 families in our community facing food shortage and to supplement national efforts as the govt continues to save us from [#COVID](https://twitter.com/hashtag/COVID?src=hashtag_click)-19.  Let’s pray we do not have a food shortage. With all the COVID-19 cases in meat plants and some other food plants like Kraft/Heinz in the coming months who knows.  Stores are cleaner and the people who work there are friendlier.  I’m in Alabama, and our Publix is the same way. Wiping down carts, limiting numbers, 1 way aisles, one way in, one way out, 6 ft. between carts, and a shield b/t cashier and the line. And most ppl are wearing masks, but not always wearing them right...  Yes! LongosMarkets requires all customers to wear masks. Went there today, it was a good, safe shopping experience, better than any other store. Will definitely be shopping there again. |
| Panic buying | “2020 and panic buying has reduced us to this. Waiting in line for at least 2 hours to get pull ups and baby wipes because no one else has them.  Screw off with all your panic buying. I actually need toilet paper now AND I CAN'T FIND IT BECAUSE YOU ALL BUY IT LIKE ITS GOING TO PROTECT YOU FROM THIS NONSENSE.  “No panic buying y’all hear that? So leave some damn bread and milk for me please”  I went shopping yesterday evening. Now there are glass screens between staff and customers. But at least there is much less panic buying. Supermarket staff are making a great effort. I foolishly feel nervous going into a place they are all day.  I used to love to go shopping, now it infuriates me! stand in line on the street for 45 minutes to get inside to tesco and not find what you need?  COVID-19 has created an entire generation of TP hoarders. We’ll all be triggered on shopping trips and buy extra...just in case...for the rest of our lives. Our children will mock us |
| Employment | I lost my job due to coronavirus. we are a local business and had to shut our doors indefinitely  Well....I just got the call. Lost my job due to Covid.....  Lost my job a couple weeks ago due to Coronavirus and now it’s impossible to find a new job  I have been laid off and my wife is a school teacher in Ohio so who knows when she will get paid or if she will again  Family of 3 Officially homeless I hate covid 19! It's caused me to lose my job, my home. Trying to get back on our feet! |
| Stock markets | Four stock market crashes in 22 years: • Asian Crisis 1997-98 • Tech Crash 2000-03 • GFC 2007-09 • Corona Virus 2020-?? Most markets around the world haven't made any gains in 13 to 20 years.  More worried about stock market crash,serious recession,business going skint, mass unemployment, ,this is a bigger threat than a virus which could kill only a fraction of the Flu this year  Stock market is going mad for Covid stocks, some genuinely good ones but some just trying to catch the wave.  No previous infectious disease outbreak... has impacted the  stock market as powerfully as the COVID-19 pandemic. ... policy responses to the COVID-19 pandemic provide the most compelling explanation for its unprecedented stock market impact  Do you think the stock market has actually priced in covid, or are we in for some crazy drops ahead? Call me old fashioned, but I thought when people can't work or buy stuff, companies aren't worth as much  I expect the US stock market to post negative annualized returns for at least the next decade  1 in ten Americans are now unemployed.The stock market still 5k down from February. There is no national plan to fight Covid. |
| Businesses | The company I work for has had to close down as a business until further notice because of the Coronavirus.  Small business retail was struggling before the COVID19 pandemic. This sudden halt will force many of our favorite Main Street shops and restaurants to close their doors forever.  I have 2 daughters that are business owners & had to close their doors. Everyone is and/or knows others being devastated by this lockdown.  How appalling that Clarks, a company with a 1.46 billion pound turnover, is forcing staff to take personal holiday allowance when stores close. Absolutely disgusting  This local sports store in Indiana was forced to close and let go of their employees after 40 years in business due to the coronavirus. But thanks to a PaycheckProtectionProgram loan, they are back in business and ten Americans have their paychecks back!  While many people (including me) and businesses are excited about starting to open again, let’s be careful.  So happy to see our Michigan greenhouses open for business. They should have never been shutdown. Support your local greenhouses!  Ok. We have had all retail, even malls open. Some of them have closed tough, no traffic, apart garden stores etc. Now people are on the move. Only restaurant closure here in Finland. Obidient punch.  Just came back from shopping in Nashville Stores were packed! Traffic back too.. People everywhere,spending money Shops, restaurants open for business People have figured it out America’s not just coming back...it will come ROARING back!  Most of HK open for business now. Emphasis on testing and tracing.  Open for business. Trusting the people to take care of themselves. Freedom smells sweet. |
| Hospitals and Clinics | Madrid hospitals now have double the number of intensive care patients than beds. Means you can no longer get intensive care in a Madrid hospital......  Very concerning with respect to lack of adequate ICU beds. Mortality might be higher not directly because of Corona but because of lack of resources  Wait for Covid-19 hospital bed now 3 days in some wards  Lack of safety gear for healthcare workers, shortage of beds and doctors, inadequate labs to conduct tests - our healthcare system is very fragile!!  Detroit's $9 million field hospital shuts after treating 39 COVID patients \| Bridge Magazine You have to be kidding me. Dems sure know how to waste taxpayer time, resources & money.  Bengaluru North Hospital messes up a COVID case. Tries to hide a case of admission, patient dies and when tested surreptitiously in Hyderabad lab (lab technician carries sample).  Thousands more will die not from Covid19 but because of the breakdown it’s causing in our healthcare system. My wife, a hospital worker in wound care, has had the hospital turn away patients who the Dr wanted admitted.  Stay at home was all about flattening curve and allowing hospitals to treat Covid. Hospital Bed availability is 80-95%. No one that has needed a bed or ventilator has been denied one. More cases are normal with 50% rise in testing.  Gandhi Nagar hospital got converted into COVID-19 hospital  CM Sindh has announced to convert the Karachi Expo Center into a 10000-Bed Hospital immediately. |
| Frontline workers | The knighted geniuses at the top of the NHS can’t even organise protective equipment for our doctors and nurses  Doctors out here are contracting the virus due to the shortage of PPE and here they are..gifting each other masks and coveralls..”  NHS staff are tearing up curtains to use as PPE. We have no tests in place. We have a shortage of ventilators. Frontline staff are dying because they don’t have equipment to keep them safe.  Three nurses forced to wear bin bags because of PPE shortage ‘test positive for coronavirus’  Really sad to see this video of the medical staff of Ambedkar Hospital,who are tested positive for Covid-19 and are struggling for proper facilities. Our Coronawarriors deserve better treatment.  Busy 1st day relieving the frontline , finished the day off at hospital with mental health apprehensive, great nurses and doctor’s working hard during Covid_19  my wife is a nurse works in case management. she gave a covid patient a ride home from the hospital today. she also went to the pharmacy to get his perscriptions and groceries for the week. just wanted to let the world know how great my wife is, and all nurses are.  After Covid-19 brokedown, thousands of people who r called our unsung heroes have been on duty since day 1 Like Doctors, policemen, Delivery guys etc. Such people r being called CORONA WARRIORS across the Nation. Your work deserves appreciation :)  I just heard, a doctor of Nepalese origin in the US has been tested positive for Corona. I wish the doctor speedy recovery. This new virus can infect us all, stay home. I will keep the name confidential respecting privacy. Hugh respect to the frontline health care professionals    Deepest gratitude to the #CoronaWarriors who are working tirelessly in these difficult times |
| Health policy | My local hospital released a statement a day ago stating only people that are 65+ or high risk can get tested due to "regional and national shortage" of tests.  The ventilator situation is even more dire than we know. Not every hospital had an allocation policy in place .  Utterly shameful if a family member can’t get a ventilator because of this policy.  Spain has begun a no ventilator policy for anyone over 65.  In NYC, (the Bronx), the nursing homes are not permitted to test. My friend’s elderly mother with Alzheimer’s is suspected of having Covid. She can only get tested at a hospital, a risk the family isn’t willing to take. Nursing homes should have mandatory onsite testing. Period.  My husband is a dialysis patient. Our state ventilator triage policy will exclude him from a vent if they are low (not even OUT) of vents  Italy has had to place a policy that ‘no one over age 60 gets a ventilator’.  Covid-19 patients are being discharged from hospital INTO CARE HOMES - many of them while still infectious. This policy means that that Covid-19 is being INTRODUCED into an environment full of extremely vulnerable people. This is murder.  Several hospitals have changed PPE policy from conservative (NO masks in hospital, no N95 for most COVID patients) to cautious (masks for EVERYONE in hospital, N95 for all COVID + PUI). So then why can’t they admit their old PPE policies were sub-optimal?  SOME hospitals are implementing a “no visitors” policy with very very few exceptions. End-of-life patients can have a one time visit, 2 visitors for 1 hour, visitation.  Relative going in for major cancer surgery next week. Spouse can’t visit under hospital’s no visitation policy. Psychologically excruciating but family all recognize it’s the right thing, and hard to limit exceptions once you start.  The hospital has an understandable policy during this crisis of limiting visitation for the safety of all & to reduce use of critical PPEs |
| Travel restrictions | There was no travel ban, it was a weak travel restriction -- 430,000 people arrived in the US from China during his "travel ban."  Just as with his Europe travel ban, this would have worked... 2 months ago. The virus is everywhere and there is community spread. Limiting travel further will do nothing to improve the situation.  Travel restriction and extensive screening at points of entry was an essential part of Taiwan's response to Covid 19. We did a half-hearted travel restriction, no screening whatsoever, and wasted what little time it bought.  Travel ban good, yes. Everything else, idiotic.  I mean the travel ban is good I think personally to try control it abit more but I also think we need to be doing SO much more as a country than what they’re imposing  I just heard a travel ban has been placed. Good one.  I think this travel ban on international flights is a very good decision. I strongly support it despite that for a while I was thinking to go back to Pakistan.  I believe it was a good move from India to have a complete travel restriction to all countries. When we don't have the health systems to treat huge populations, the best thing to do is to shut doors  The fine for breaking self-quarantine / self-isolation in BC, Canada is $25,000 AND jail time. Canada is taking travel and quarantine very seriously. Great job. |
| Lockdowns | France extends Covid-19 lockdown beyond 15 April  China has imposed NEW coronavirus lockdown - fears grow for devastating second wave  Extraordinary video out of Indian about how the lockdown is impacting day laborers. I was only able to watch 2 minutes because it is so awful. The lockdown was imposed with 4 hours notice. People are beginning to starve already.  It appears the lockdown in Nigeria has some impact. In the two weeks before the lockdown, the average daily increase in new Covid 19 cases was around 42%. Since the start of the lockdown, the average daily increase has been around 4%. We need to be patient and do the needful  New Zealand's prime minister introduced a brutal level-4 restriction lockdown after the first few cases of Covid-19. They are practically over the epidemic now with only 1,200 cases and one death.  I think the Curfew should be extended for 15 days from now. All possible cases will come out. The spread will be contained. Also whoever is being brought from abroad should be sent for mandatory quarantine for a month. Travel restrictions should continue for Atleast 2 month. |
| Financial Measures | My family took a 60% pay decrease when we moved back to the US due to COVID, we haven’t got our taxes back, nor are we getting our stimulus...  Corporations steal most of the COVID stimulus package, intended for small businesses and working-class people  I just received my stimulus check. I’ve been lucky enough to get a new job.  my daughter phoned me up this morning and informed me she got a $1200 Covid stimulus check from the USA. Apparently US residency isn't a requirement - just filing US taxes. I guess one is probably coming for me too then. Bonus  I got my stimulus check today! Woohoo!  Zoe and I finally received our stimulus/relief check from the federal government |
| Impact on politics | While Filthy and Despicable Democrats are blaming President Trump for the spread of Chinese Virus, I just want to remind everyone that Democrats unilaterally called him a Xenophobe for putting a travel ban on China and Europe in the early stages of this Pandemic  Democrats want to steal the 2020 election while everyone is quarantined and distracted by the virus.  this virus, the whole election thing, trump, school... everything’s like hella overwhelming right now.  Why are you using the virus to run an election campaign? It's disgusting.  Covid-19 is agenda of next election. Some parties will promise we will never allow CORONAVIRUS spread.  Using COVID-19 as a voter suppression tool is unAmerican. To have a fair election in November, we MUST expand vote by mail and early voting. |
| Racism | This Chinese virus, damn China destroyed whole world. May God curse China!  Chinese virus is killing our people!!! Chinese Citizens are mistreating our people!!!Ata sisi we should mistreat them [http://too.an](https://t.co/tbCyl3nslH?amp=1) eye for an eye  President was right. This a Chinese virus manufactured in a lab in Wuhan. Chinese want to infect everyone in the world by force.  It is Chinese virus incubated and produced due to dirty Chinese food habits.  Why can’t we call Chinese Virus like calling a spade a spade?  Y'all see these Chinese are racist AF, but act like victims when the spotlight is on them its the Chinese virus and no body can tell me sh*t CNN stop trump agenda and deliver real news, y'all were all anti trump when he says Chinese virus but silent on China saying foreign virus  Well if the Chinese weren’t eating bats & dogs & whatever else from the Wuhan Meat Market then we wouldn’t have this problem |
